# Supplementary material for: Changes in gray whale phenology and distribution related to prey variability and ocean biophysics in the northern Bering and eastern Chukchi seas
Source: PLoS One. 2022 Apr 7;17(4):e0265934. doi: 10.1371/journal.pone.0265934 (PMC8989348; doi:10.1371/journal.pone.0265934)
Supplement: S1 Table — (PDF) [file pone.0265934.s001.pdf]

Table S-1. DBO cruises where a marine mammal watch was conducted, 2009-2019. Xs - indicate number of cruises/year on that ship; *RUSALCA cruises were on either the *Professor Khromov*, or Norseman 2. Metadata, ship track and sightings available at NSF Arctic Data Center

Ship/year	2009	2010	2011	2012	2013	2014	2015	2016	2017	2018	2019	Total per ship
Healy				X	X			X	X	X		5
*RUSALCA	X	X	X	X	X							5
Norseman 2							X		X			2
Sikuliaq									XX	X		3
SWL			X		X	X	X	X		X	X	7
Total per year	1	1	2	2	3	1	2	2	4	3	1	22
