# Supplementary material for: Changes in gray whale phenology and distribution related to prey variability and ocean biophysics in the northern Bering and eastern Chukchi seas
Source: PLoS One. 2022 Apr 7;17(4):e0265934. doi: 10.1371/journal.pone.0265934 (PMC8989348; doi:10.1371/journal.pone.0265934)
Supplement: S2 Table — (PDF) [file pone.0265934.s002.pdf]

Table S-2. Gray whale sighting rates derived from ASAMM data for the Hope Basin, Wainwright and Peard Bay analytical areas. Excludes sightings and effort from coastal transect.

Hope Basin Area	July			August			September			Total		
Year	km	GW	SR	km	GW	SR	km	GW	SR	Km	GW	SR
2009	0	0	NA	0	0	NA	0	0	NA	0	0	NA
2010	81	7	0.0864	85	1	0.0118	0	0	NA	166	8	NA
2011	81	2	0.0247	80	1	0.0125	79	1	0.0127	240	4	0.0167
2012	0	0	NA	0	0	NA	78	0	0.0000	78	0	0.0000
2013	80	2	0.0250	96	2	0.0208	64	0	0.0000	240	4	0.0167
2014	407	7	0.0172	436	77	0.1766	435	44	0.1011	1278	128	0.1002
2015	0	0	NA	406	6	0.0148	48	0	0.0000	454	6	0.0132
2016	299	16	0.0535	590	26	0.0441	484	90	0.1860	1373	132	0.0961
2017	284	67	0.2359	424	69	0.1627	431	18	0.0418	1139	154	0.1352
2018	719	66	0.0918	61	0	0.0000	498	4	0.0080	1278	70	0.0548
2019	284	42	0.1479	411	3	0.0073	441	28	0.0635	1136	73	0.0643
Total	2235	209	0.0935	2589	185	0.0715	2558	185	0.0723	7382	579	0.0784

Wainwright Area	July			August			September			Total		
Year	km	GW	SR	km	GW	SR	km	GW	SR	km	GW	SR
2009	671	23	0.0343	407	12	0.0295	614	7	0.0114	1692	42	0.0248
2010	564	8	0.0142	430	13	0.0302	682	1	0.0015	1676	22	0.0131
2011	346	4	0.0116	766	19	0.0248	721	15	0.0208	1833	38	0.0207
2012	856	27	0.0315	667	19	0.0285	706	9	0.0127	2229	55	0.0247
2013	662	20	0.0302	875	9	0.0103	632	13	0.0206	2169	42	0.0194
2014	531	13	0.0245	471	7	0.0149	356	1	0.0028	1358	21	0.0155
2015	515	45	0.0874	828	26	0.0314	869	8	0.0092	2212	79	0.0357
2016	795	65	0.0818	994	36	0.0362	840	24	0.0286	2629	125	0.0475
2017	874	68	0.0778	614	17	0.0277	936	34	0.0363	2424	119	0.0491
2018	664	85	0.1280	697	34	0.0488	922	101.4	0.1100	2283	220.4	0.0965
2019	417	44	0.1055	46	3	0.0652	909	28	0.0308	1372	75	0.0547
Total	6895	402	0.0583	6795	195	0.0287	8187	241.4	0.0295	21877	838.4	0.0383
Peard Bay Area	July			August			September			Total		
Year	km	GW	SR	km	GW	SR	km	GW	SR	km	GW	SR
2009	418	10	0.0239	510	38	0.0745	442	9	0.0204	1370	57	0.0416

2010	437	28	0.0641	305	8	0.0262	400	2	0.0050	1142	38	0.0333
2011	411	28	0.0681	418	13	0.0311	901	23	0.0255	1730	64	0.0370
2012	763	15	0.0197	593	23	0.0388	628	6	0.0096	1984	44	0.0222
2013	464	3	0.0065	720	24	0.0333	591	5	0.0085	1775	30	0.0169
2014	495	11	0.0222	517	16	0.0309	430	7	0.0163	1442	34	0.0236
2015	472	25	0.0530	758	25	0.0330	1130	6	0.0053	2360	56	0.0237
2016	705	17	0.0241	441	3	0.0068	903	17	0.0188	2049	37	0.0181
2017	796	59	0.0741	744	0	0.0000	1085	6	0.0055	2625	65	0.0248
2018	796	9	0.0113	441	0	0.0000	936	5	0.0053	2173	14	0.0064
2019	591	4	0.0068	289	0	0.0000	1106	0	0.0000	1986	4	0.0020
Total	6348	207	0.0326	5736	150	0.0262	8552	86	0.0101	20636	443	0.0215
