# Supplementary material for: Changes in gray whale phenology and distribution related to prey variability and ocean biophysics in the northern Bering and eastern Chukchi seas
Source: PLoS One. 2022 Apr 7;17(4):e0265934. doi: 10.1371/journal.pone.0265934 (PMC8989348; doi:10.1371/journal.pone.0265934)
Supplement: S3 Table — (PDF) [file pone.0265934.s003.pdf]

Table S-3. Crustacea abundance by year and station number.

DBO region	Cruise ID	Station Number	Station Name	Date	Data Year	Latitude (°N)	Longitude (°W)	Average Abundance for Class Crustacea (no/m2)
2	SWL2010	20	UTBS5	20100717	2010	64.671	-169.92	1243
2	SWL2010	21	UTBS2	20100717	2010	64.682	-169.1	15653
2	SWL2010	22	UTBS4	20100718	2010	64.958	-169.88	200
2	SWL2010	23	UTBS1	20100718	2010	64.991	-169.14	14070
2	SWL2011	8	UTBS5	20110716	2011	64.670	-169.92	1413
2	SWL2011	9	UTBS1	20110716	2011	64.990	-169.14	19445
2	SWL2011	10	UTBS2	20110716	2011	64.680	-169.100	19643
2	SWL2011	11	UTBS4	20110716	2011	64.960	-169.89	108
2	SWL2012	10	UTBS4	20120714	2012	64.961	-169.89	700
2	SWL2012	7	UTBS5	20120716	2012	64.669	-169.92	1240
2	SWL2012	8	UTBS2	20120716	2012	64.680	-169.1	15553
2	SWL2012	9	UTBS1	20120716	2012	64.991	-169.14	25280
2	SWL2013	14	UTBS5	20130715	2013	64.671	-169.92	665
2	SWL2013	15	UTBS4	20130715	2013	64.963	-169.89	910
2	SWL2013	16	UTBS2	20130715	2013	64.683	-169.1	3008
2	SWL2013	18	UTBS1	20130715	2013	64.992	-169.14	11320
2	SWL2014	14	UTBS5	20140715	2014	64.671	-169.92	845
2	SWL2014	15	UTBS2	20140716	2014	64.682	-169.10	5548
2	SWL2014	17	UTBS4	20140716	2014	64.960	-169.89	538
2	SWL2014	18	UTBS1	20140716	2014	64.991	-169.14	15785
2	SWL2015	14	UTBS5	20150715	2015	64.672	-169.92	2040
2	SWL2015	15	UTBS2	20150716	2015	64.682	-169.11	5460
2	SWL2015	17	UTBS4	20150716	2015	64.961	-169.89	165
2	SWL2015	18	UTBS1	20150716	2015	64.991	-169.14	19635
2	SWL2016	92	UTBS2A	20160714	2016	64.669	-168.23	1633
2	SWL2017	71	BCL6C	20170715	2017	64.672	-170.64	725
2	SWL2017	80	UTBS5	20170716	2017	64.670	-169.91	1680
2	SWL2017	86	UTBS2	20170716	2017	64.681	-169.1	3950
2	SWL2017	92	UTBS2A	20170716	2017	64.671	-168.24	2663
2	SWL2017	97	DBO2.7	20170716	2017	65.001	-168.22	3485
2	SWL2017	103	UTBS1	20170716	2017	64.990	-169.14	15403
2	SWL2017	109	UTBS4	20170716	2017	64.961	-169.89	438
2	SWL2018	75	BCL6C	20180718	2018	64.673	-170.64	1393
2	SWL2018	82	UTBS5	20180718	2018	64.673	-169.93	1365
2	SWL2018	89	UTBS2	20180718	2018	64.681	-169.1	6998
2	SWL2018	95	UTBS2A	20180718	2018	64.672	-168.24	3430
2	SWL2018	101	DBO2.7	20180718	2018	65.000	-168.22	4578
2	SWL2018	108	UTBS1	20180718	2018	64.992	-169.14	8633
2	SWL2018	115	UTBS4	20180718	2018	64.960	-169.89	488
2	SWL2019	90	BCL6c	20190715	2019	64.672	-170.64	2063

2	SWL2019	97	UTBS5	20190715	2019	64.671	-169.92	2270
2	SWL2019	104	UTBS2	20190715	2019	64.681	-169.1	6145
2	SWL2019	111	UTBS2A	20190716	2019	64.669	-168.23	1268
2	SWL2019	118	UTBS1	20190716	2019	64.990	-169.14	5333
2	SWL2019	125	UTBS4	20190716	2019	64.961	-169.89	210
2	SWL2016	74	BCL6C	20160714	2016	64.673	-170.64	1580
2	SWL2016	80	UTBS5	20160714	2016	64.671	-169.92	805
2	SWL2016	86	UTBS2	20160714	2016	64.680	-169.1	5673
2	SWL2016	97	DBO2.7	20160714	2016	65.000	-168.22	3835
2	SWL2016	104	UTBS1	20160714	2016	64.991	-169.14	15953
2	SWL2016	110	UTBS4	20160715	2016	64.962	-169.89	383
3	SWL2010	29	UTN1	20100718	2010	66.707	-168.4	608
3	SWL2010	30	UTN2	20100719	2010	67.048	-168.73	200
3	SWL2010	31	UTN3	20100719	2010	67.335	-168.96	143
3	SWL2010	32	UTN4	20100719	2010	67.501	-168.91	378
3	SWL2010	33	JTN5=SEC1	20100719	2010	67.669	-168.96	1000
3	SWL2010	34	UTN6	20100719	2010	67.736	-168.44	243
3	SWL2010	35	UTN7	20100719	2010	68.004	-168.92	6208
3	SWL2011	13	UTN1	20110717	2011	66.710	-168.4	380
3	SWL2011	14	UTN2	20110717	2011	67.050	-168.73	2123
3	SWL2011	15	UTN3	20110717	2011	67.330	-168.91	1248
3	SWL2011	16	UTN4	20110717	2011	67.500	-168.91	2440
3	SWL2011	17	JTN5=SEC1	20110717	2011	67.670	-168.91	5785
3	SWL2011	18	UTN6	20110718	2011	67.740	-168.44	5570
3	SWL2011	19	SEC2	20110718	2011	67.780	-168.6	6955
3	SWL2011	20	UTN7	20110718	2011	68.000	-168.91	10820
3	SWL2011	21	SEC3	20110718	2011	67.900	-168.24	4945
3	SWL2011	22	SEC4	20110718	2011	68.010	-167.87	4435
3	SWL2011	23	SEC5	20110718	2011	68.130	-167.5	1030
3	SWL2011	24	SEC6	20110718	2011	68.190	-167.31	45
3	SWL2011	25	SEC7	20110718	2011	68.240	-167.12	45
3	SWL2011	26	SEC8	20110718	2011	68.301	-166.94	15
3	SWL2012	12	UTN1	20120714	2012	66.712	-168.4	223
3	SWL2012	13	UTN2	20120714	2012	67.050	-168.73	610
3	SWL2012	14	UTN3	20120714	2012	67.329	-168.91	3670
3	SWL2012	15	UTN4	20120715	2012	67.500	-168.9	3535
3	SWL2012	16	JTN5=SEC1	20120715	2012	67.670	-168.91	4100
3	SWL2012	17	UTN6	20120715	2012	67.740	-168.44	5465
3	SWL2012	18	SEC2	20120715	2012	67.780	-168.6	4405
3	SWL2012	19	UTN7	20120716	2012	68.001	-168.91	4270
3	SWL2012	20	SEC3	20120716	2012	67.901	-168.24	10490
3	SWL2012	21	SEC4	20120716	2012	68.010	-167.87	1260
3	RUSALCA12	6	SEC8=cs17	20120901	2012	68.298	-167.04	785
3	RUSALCA12	54	SEC3=cs12r	20120915	2012	67.860	-168.34	7863
3	SWL2013	21	UTN1	20130719	2013	66.711	-168.4	290

3	SWL2013	22	UTN2	20130719	2013	67.050	-168.73	835
3	SWL2013	23	UTN3	20130719	2013	67.328	-168.94	1708
3	SWL2013	24	UTN4	20130719	2013	67.500	-168.94	6748
3	SWL2013	25	JTN5=SEC1	20130719	2013	67.670	-168.94	5605
3	SWL2013	26	UTN6	20130719	2013	67.742	-168.44	10778
3	SWL2013	27	SEC2	20130719	2013	67.779	-168.61	9623
3	SWL2013	32	SEC4	20130720	2013	68.014	-167.87	1018
3	SWL2013	33	SEC3	20130720	2013	67.898	-168.23	9145
3	SWL2013	34	UTN7	20130720	2013	67.999	-168.94	13053
3	SWL2014	20	UTN1	20140717	2014	66.712	-168.4	338
3	SWL2014	21	UTN2	20140717	2014	67.050	-168.73	118
3	SWL2014	22	UTN3	20140717	2014	67.330	-168.91	688
3	SWL2014	23	UTN4	20140717	2014	67.501	-168.9	635
3	SWL2014	24	UTN6	20140717	2014	67.741	-168.44	1940
3	SWL2014	25	SEC8	20140718	2014	68.300	-166.94	80
3	SWL2014	26	SEC7	20140718	2014	68.242	-167.12	180
3	SWL2014	27	SEC6	20140718	2014	68.185	-167.31	240
3	SWL2014	28	SEC5	20140718	2014	68.128	-167.5	1040
3	SWL2014	29	SEC4	20140718	2014	68.012	-167.87	1783
3	SWL2014	30	SEC3	20140718	2014	67.898	-168.23	6215
3	SWL2014	31	SEC2	20140718	2014	67.783	-168.6	6218
3	SWL2014	32	JTN5=SEC1	20140718	2014	67.671	-168.91	2213
3	SWL2014	33	UTN7	20140719	2014	68.000	-168.93	12873
3	SWL2015	20	UTN1	20150716	2015	66.711	-168.4	235
3	SWL2015	21	UTN2	20150717	2015	67.050	-168.73	178
3	SWL2015	22	UTN3	20150717	2015	67.328	-168.94	568
3	SWL2015	23	UTN4	20150717	2015	67.500	-168.94	1273
3	SWL2015	24	SEC8	20150717	2015	68.302	-166.94	0
3	SWL2015	25	SEC7	20150717	2015	68.242	-167.12	350
3	SWL2015	26	SEC6	20150717	2015	68.185	-167.31	1410
3	SWL2015	27	SEC5	20150717	2015	68.127	-167.5	560
3	SWL2015	28	SEC4	20150718	2015	68.013	-167.87	795
3	SWL2015	29	SEC3	20150718	2015	67.898	-168.23	7225
3	SWL2015	30	UTN6	20150718	2015	67.740	-168.44	7545
3	SWL2015	31	SEC2	20150718	2015	67.783	-168.6	9510
3	SWL2015	32	JTN5=SEC1	20150718	2015	67.670	-168.96	5588
3	SWL2015	33	UTN7	20150718	2015	68.000	-168.94	39858
3	SWL2017	126	UTN2	20170717	2017	67.050	-168.73	333
3	SWL2017	131	UTN3	20170717	2017	67.331	-168.91	1658
3	SWL2017	136	UTN4	20170717	2017	67.500	-168.91	1410
3	SWL2017	142	SEC8	20170718	2017	68.297	-166.94	820
3	SWL2017	147	SEC7	20170718	2017	68.243	-167.12	710
3	SWL2017	152	SEC6	20170718	2017	68.185	-167.31	1600
3	SWL2017	157	SEC5	20170718	2017	68.128	-167.5	1360
3	SWL2017	162	SEC4	20170718	2017	68.013	-167.87	1083

3	SWL2017	168	SEC3	20170718	2017	67.898	-168.23	9550
3	SWL2017	174	UTN6	20170719	2017	67.741	-168.43	8735
3	SWL2017	179	SEC2	20170719	2017	67.783	-168.6	13828
3	SWL2017	184	JTN5=SEC1	20170719	2017	67.675	-168.92	2393
3	SWL2017	189	UTN7	20170719	2017	68.001	-168.93	19508
3	HLY1702	1	JTN5=SEC1	20170829	2017	67.677	-168.95	1440
3	HLY1702	2	SEC2	20170829	2017	67.783	-168.6	11078
3	HLY1702	3	SEC3	20170829	2017	67.898	-168.23	10163
3	HLY1702	4	SEC4	20170829	2017	68.013	-167.88	1820
3	HLY1702	5	SEC5	20170830	2017	68.133	-167.49	1630
3	HLY1702	6	SEC6	20170830	2017	68.187	-167.3	800
3	HLY1702	7	SEC7	20170830	2017	68.247	-167.12	190
3	HLY1702	8	SEC8	20170830	2017	68.304	-166.92	520
3	SWL2018	127	UTN1	20180719	2018	66.709	-168.4	218
3	SWL2018	134	UTN2	20180719	2018	67.050	-168.73	418
3	SWL2018	141	UTN3	20180719	2018	67.331	-168.91	1040
3	SWL2018	148	UTN4	20180720	2018	67.500	-168.91	1078
3	SWL2018	155	SEC8	20180720	2018	68.299	-166.94	750
3	SWL2018	161	SEC7	20180720	2018	68.244	-167.12	790
3	SWL2018	167	SEC6	20180720	2018	68.186	-167.31	680
3	SWL2018	173	SEC5	20180720	2018	68.128	-167.49	1740
3	SWL2018	179	SEC4	20180720	2018	68.013	-167.87	1370
3	SWL2018	186	SEC3	20180721	2018	67.899	-168.24	5345
3	SWL2018	193	UTN6	20180721	2018	67.740	-168.44	3678
3	SWL2018	200	SEC2	20180721	2018	67.784	-168.6	3870
3	SWL2018	207	JTN5=SEC1	20180721	2018	67.672	-168.93	1763
3	SWL2018	214	UTN7	20180721	2018	68.000	-168.93	3413
3	SWL2019	136	UTN1	20190717	2019	66.708	-168.4	110
3	SWL2019	142	UTN2	20190717	2019	67.050	-168.73	213
3	SWL2019	149	UTN3	20190717	2019	67.331	-168.91	1578
3	SWL2019	155	UTN4	20190717	2019	67.500	-168.91	1000
3	SWL2019	162	UTN6	20190717	2019	67.739	-168.44	1178
3	SWL2019	169	SEC8	20190718	2019	68.299	-166.94	200
3	SWL2019	175	SEC7	20190718	2019	68.243	-167.12	220
3	SWL2019	181	SEC6	20190718	2019	68.184	-167.31	1930
3	SWL2019	187	SEC5	20190718	2019	68.130	-167.5	1240
3	SWL2019	193	SEC4	20190718	2019	68.012	-167.86	720
3	SWL2019	199	SEC3	20190718	2019	67.896	-168.23	1940
3	SWL2019	206	SEC2	20190718	2019	67.782	-168.6	1683
3	SWL2019	213	SEC1	20190719	2019	67.671	-168.93	2053
3	SWL2019	220	UTN7	20190719	2019	68.001	-168.93	2090
3	SWL2016	121	UTN1	20160715	2016	66.710	-168.4	288
3	SWL2016	126	UTN2	20160715	2016	67.050	-168.73	158
3	SWL2016	132	UTN3	20160716	2016	67.331	-168.95	1050
3	SWL2016	137	UTN4	20160716	2016	67.500	-168.91	1205

3	SWL2016	142	SEC8	20160716	2016	68.299	-166.94	240
3	SWL2016	147	SEC7	20160716	2016	68.245	-167.12	310
3	SWL2016	152	SEC6	20160716	2016	68.186	-167.31	1340
3	SWL2016	158	SEC5	20160716	2016	68.130	-167.49	1360
3	SWL2016	163	SEC4	20160717	2016	68.013	-167.87	855
3	SWL2016	168	SEC3	20160717	2016	67.899	-168.23	9490
3	SWL2016	173	UTN6	20160717	2016	67.738	-168.43	11247
3	SWL2016	178	SEC2	20160717	2016	67.783	-168.6	14588
3	SWL2016	183	JTN5=SEC1	20160717	2016	67.671	-168.96	5807
3	SWL2016	188	UTN7	20160717	2016	68.001	-168.93	30135
4	SWL2013	35	DBO4.6	20130721	2013	71.615	-163.79	303
4	SWL2013	36	DBO4.5	20130721	2013	71.491	-163.41	1315
4	SWL2013	37	DBO4.4	20130722	2013	71.360	-163.03	2080
4	SWL2013	38	DBO4.3	20130722	2013	71.232	-162.64	2648
4	SWL2013	39	DBO4.2	20130722	2013	71.103	-162.28	4723
4	SWL2013	40	DBO4.1	20130722	2013	70.973	-161.9	7373
4	SWL2014	34	DBO4.1	20140720	2014	70.973	-161.9	5705
4	SWL2014	36	DBO4.2	20140720	2014	71.104	-162.26	1460
4	SWL2014	38	DBO4.3	20140720	2014	71.233	-162.64	3925
4	SWL2014	40	DBO4.4	20140721	2014	71.362	-163.01	2860
4	SWL2014	42	DBO4.5	20140721	2014	71.490	-163.39	1173
4	SWL2014	44	DBO4.6	20140721	2014	71.618	-163.77	768
4	SWL2015	34	DBO4.6	20150719	2015	71.617	-163.79	278
4	SWL2015	36	DBO4.5	20150719	2015	71.490	-163.41	1565
4	SWL2015	38	DBO4.4	20150719	2015	71.362	-163.03	3178
4	SWL2015	40	DBO4.3	20150720	2015	71.233	-162.65	2250
4	SWL2015	42	DBO4.2	20150720	2015	71.104	-162.27	3363
4	SWL2015	44	DBO4.1	20150720	2015	70.974	-161.9	5203
4	\MBON2015	29	)4.1n=ML6	20150819	2015	71.193	-160.27	10555
4	\MBON2015	31	O4.2n=ML4	20150820	2015	71.324	-160.66	1078
4	\MBON2015	32	O4.3n=ML4	20150820	2015	71.455	-161.04	208
4	\MBON2015	33	O4.4n=ML4	20150820	2015	71.588	-161.4	120
4	\MBON2015	35	O4.5n=ML4	20150821	2015	71.719	-161.77	138
4	\MBON2015	36	O4.6n=ML4	20150821	2015	71.851	-162.16	388
4	SWL2017	194	DBO4.6	20170720	2017	71.617	-163.77	585
4	SWL2017	200	DBO4.5	20170720	2017	71.489	-163.39	990
4	SWL2017	207	DBO4.4	20170720	2017	71.363	-163.01	1600
4	SWL2017	213	DBO4.3	20170721	2017	71.231	-162.64	6470
4	SWL2017	219	DBO4.2	20170721	2017	71.104	-162.26	1360
4	SWL2017	225	DBO4.1	20170721	2017	70.973	-161.9	10550
4	HLY1702	10	DBO4.6	20170831	2017	71.620	-163.76	1650
4	HLY1702	12	DBO4.5	20170831	2017	71.490	-163.38	690
4	HLY1702	14	DBO4.4	20170831	2017	71.364	-163	2730
4	HLY1702	16	DBO4.3	20170831	2017	71.233	-162.64	4950
4	HLY1702	18	DBO4.2	20170831	2017	71.102	-162.25	11640

4	HLY1702	20	DBO4.1	20170831	2017	70.975	-161.89	4190
4	HLY1702	123	BO4.6N=SE	20170909	2017	71.774	-161.6	108
4	HLY1702	126	)BO4.6N=S:	20170909	2017	71.610	-161.63	190
4	HLY1702	128	)BO4.5N=S:	20170910	2017	71.479	-161.52	490
4	HLY1702	129	)BO4.4N=S:	20170910	2017	71.349	-161.41	400
4	HLY1702	130	)BO4.3N=S4	20170910	2017	71.221	-161.3	7320
4	HLY1702	131	)BO4.2N=S!	20170910	2017	71.089	-161.2	29468
4	HLY1702	132	)BO4.1N=S(	20170910	2017	70.961	-161.09	2300
4	SWL2018	223	DBO4.6n	20180722	2018	71.852	-162.16	225
4	SWL2018	230	DBO4.5n	20180722	2018	71.743	-161.85	125
4	SWL2018	238	DBO4.4n	20180722	2018	71.588	-161.4	55
4	SWL2018	246	DBO4.3n	20180722	2018	71.454	-161.04	115
4	SWL2016	193	DBO4.1	20160718	2016	70.973	-161.9	9505
4	SWL2016	199	DBO4.2	20160718	2016	71.101	-162.27	12953
4	SWL2016	204	DBO4.3	20160719	2016	71.235	-162.64	2473
4	SWL2016	209	DBO4.6	20160720	2016	71.618	-163.78	370
5	COMIDA10	50	3arc5=CBL9	20100812	2010	71.414	-157.49	8103
5	SWL2011	28	BarC10	20110720	2011	71.620	-157.93	110
5	SWL2011	29	BarC9	20110720	2011	71.580	-157.84	170
5	SWL2011	30	BarC8	20110720	2011	71.540	-157.75	205
5	SWL2011	31	BarC7	20110720	2011	71.500	-157.66	11935
5	SWL2011	32	BarC6	20110720	2011	71.460	-157.58	22835
5	SWL2011	33	BarC5	20110720	2011	71.410	-157.49	14663
5	SWL2011	34	BarC4	20110720	2011	71.370	-157.42	6665
5	SWL2011	35	BarC3	20110720	2011	71.330	-157.33	1090
5	SWL2011	36	BarC2	20110720	2011	71.290	-157.25	1900
5	SWL2011	37	BarC1	20110720	2011	71.250	-157.17	10870
5	HLY1201	53	BarC1	20120822	2012	71.245	-157.19	6920
5	HLY1201	54	BarC2	20120822	2012	71.282	-157.26	4130
5	HLY1201	55	BarC3	20120822	2012	71.305	-157.38	2820
5	HLY1201	56	BarC4	20120822	2012	71.366	-157.42	130
5	HLY1201	57	BarC5	20120822	2012	71.401	-157.54	5705
5	HLY1201	58	BarC6	20120822	2012	71.450	-157.61	27520
5	HLY1201	59	BarC7	20120822	2012	71.493	-157.68	1870
5	HLY1201	60	BarC8	20120823	2012	71.534	-157.76	440
5	HLY1201	61	BarC9	20120823	2012	71.575	-157.84	400
5	HLY1201	62	BarC10	20120823	2012	71.613	-157.92	140
5	SWL2013	41	BarC2	20130722	2013	71.291	-157.23	660
5	SWL2013	42	BarC1	20130722	2013	71.245	-157.19	7730
5	SWL2013	43	BarC3	20130723	2013	71.335	-157.29	1900
5	SWL2013	44	BarC4	20130723	2013	71.373	-157.4	1600
5	SWL2013	45	BarC5	20130723	2013	71.407	-157.47	12378
5	HLY1301	39	BarC1	20130808	2013	71.250	-157.17	3190
5	HLY1301	40	BarC2	20130808	2013	71.290	-157.25	100
5	HLY1301	41	BarC3	20130808	2013	71.330	-157.33	2380

5	HLY1301	42	BarC4	20130808	2013	71.370	-157.42	1600
5	HLY1301	43	BarC5	20130808	2013	71.413	-157.5	7443
5	HLY1301	44	BarC6	20130808	2013	71.460	-157.58	10960
5	HLY1301	45	BarC7	20130808	2013	71.500	-157.66	3550
5	HLY1301	46	BarC8	20130808	2013	71.540	-157.75	650
5	HLY1301	47	BarC9	20130808	2013	71.580	-157.84	180
5	HLY1301	48	BarC10	20130809	2013	71.620	-157.93	100
5	SWL2014	45	BarC3	20140722	2014	71.331	-157.32	6130
5	SWL2014	46	BarC4	20140723	2014	71.358	-157.36	1230
5	SWL2014	47	BarC5	20140723	2014	71.399	-157.46	15090
5	SWL2015	45	BarC10	20150720	2015	71.617	-157.91	180
5	SWL2015	46	BarC9	20150720	2015	71.578	-157.83	220
5	SWL2015	47	BarC8	20150720	2015	71.537	-157.75	440
5	SWL2015	48	BarC7	20150720	2015	71.500	-157.67	4310
5	SWL2015	49	BarC6	20150721	2015	71.456	-157.58	25530
5	SWL2015	50	BarC5	20150721	2015	71.410	-157.49	11235
5	SWL2015	51	BarC4	20150721	2015	71.372	-157.41	3650
5	SWL2015	52	BarC3	20150721	2015	71.330	-157.33	3780
5	SWL2015	53	BarC2	20150721	2015	71.288	-157.25	6900
5	SWL2015	54	BarC1	20150721	2015	71.247	-157.16	2700
5	SWL2017	256	BarC5	20170722	2017	71.410	-157.49	4510
5	HLY1702	21	BarC10	20170901	2017	71.623	-157.9	220
5	HLY1702	22	BarC9	20170901	2017	71.579	-157.81	380
5	HLY1702	23	BarC8	20170901	2017	71.537	-157.73	290
5	HLY1702	24	BarC7	20170901	2017	71.497	-157.65	2850
5	HLY1702	25	BarC6	20170901	2017	71.456	-157.57	38930
5	HLY1702	26	BarC5	20170901	2017	71.409	-157.47	6210
5	HLY1702	27	BarC4	20170901	2017	71.373	-157.39	760
5	HLY1702	28	BarC3	20170901	2017	71.327	-157.31	890
5	HLY1702	29	BarC2	20170901	2017	71.288	-157.23	4630
5	HLY1702	30	BarC1	20170901	2017	71.248	-157.15	670
5	SWL2019	290	BarC7	20190721	2019	71.500	-157.67	4600
