# Supplementary material for: Changes in gray whale phenology and distribution related to prey variability and ocean biophysics in the northern Bering and eastern Chukchi seas
Source: PLoS One. 2022 Apr 7;17(4):e0265934. doi: 10.1371/journal.pone.0265934 (PMC8989348; doi:10.1371/journal.pone.0265934)
Supplement: Attachment — Submitted filename: PONE-D-21-20781_reviewer.pdf [file pone.0265934.s004.pdf]

PLOS ONE

Title: Changes in gray whale phenology and distribution related to prey variability and ocean biophysics in the northern Bering and eastern Chukchi seas

--Manuscript Draft--

**Manuscript Number:**	PONE-D-21-20781
**Article Type:**	Research Article
**Full Title:**	Title: Changes in gray whale phenology and distribution related to prey variability and ocean biophysics in the northern Bering and eastern Chukchi seas
**Short Title:**	Changes in gray whale ecology related ocean biophysics in the northern Bering and eastern Chukchi seas
**Corresponding Author:**	Sue E. Moore, Ph.D.<br>University of Washington<br>Seattle, WA UNITED STATES
**Keywords:**	Key words: gray whale, amphipod prey, krill, transport, wind forcing, AMP model, Arctic, northern Bering Sea, Chukchi Sea
**Abstract:**	ABSTRACT<br>Changes in gray whale (Eschrichtius robustus) phenology and distribution are related to observed and hypothesized prey availability, bottom water temperature, salinity, sea ice persistence, integrated water column and sediment chlorophyll a , and patterns of wind-driven biophysical forcing in the northern Bering and eastern Chukchi Seas. This portion of the Pacific Arctic includes four Distributed Biological Observatory (DBO) sampling regions. In the Bering Strait area, passive acoustic data show gray whale phenology changed coincident with unprecedented wintertime sea ice loss there in 2017-2019, although some whales were seen in on DBO cruises. In the northern Bering Sea, changes in gray whale distribution indicate that they continue to target a shrinking field of infaunal amphipods, with a significant decrease in prey abundance (r = -0.313, p<0.05) observed in the DBO 2 region over the 2010-2019 period. In the eastern Chukchi Sea, sightings during broad scale aerial surveys show that gray whale distribution is associated with localized areas of high infaunal crustacean abundance. Although abundance was unchanged in DBO regions 3, 4 and 5, a mid-decade shift in gray whale distribution suggests that targeted prey may now regularly include epi-benthic and pelagic species. Specifically, in the northeastern Chukchi Sea, increased sighting rates (SR = whales/km) associated with an offshore shift in distribution was positively correlated with large scale and local wind patterns conducive to increased availability of krill there. In the southern Chukchi Sea, gray whale distribution clustered in all years near an amphipod-krill ‘hotspot’ associated with a 50-60m deep trough. We discuss potential impacts of observed and inferred prey shifts on gray whale nutrition in the context of an ongoing unusual gray whale mortality event. To conclude, we use the conceptual Arctic Marine Pulses (AMP) model to frame hypotheses that may guide future research on whales in the Pacific Arctic marine ecosystem.
**Order of Authors:**	Sue E. Moore, Ph.D.
	Janet T. Clarke
	Stephen R. Okkonen
	Jacqueline M. Grebmeier
	Catherine L. Berchok
	Kathleen M. Stafford
**Additional Information:**	
**Question**	**Response**
**Financial Disclosure**	Funding<br>CB - PAM acoustic data: Interagency Agreement between BOEM and AFSC

Powered by Editorial Manager® and ProduXion Manager® from Aries Systems Corporation

Enter a financial disclosure statement that describes the sources of funding for the work included in this submission. Review the submission guidelines for detailed requirements. View published research articles from *PLOS ONE* for specific examples.<br><br>This statement is required for submission and **will appear in the published article** if the submission is accepted. Please make sure it is accurate.<br><br>**Unfunded studies**<br>Enter: *The author(s) received no specific funding for this work.*<br><br>**Funded studies**<br>Enter a statement with the following details:<br>• Initials of the authors who received each award<br>• Grant numbers awarded to each author<br>• The full name of each funder<br>• URL of each funder website<br>• Did the sponsors or funders play any role in the study design, data collection and analysis, decision to publish, or preparation of the manuscript?<br>• **NO** - Include this sentence at the end of your statement: *The funders had no role in study design, data collection and analysis, decision to publish, or preparation of the manuscript.*<br>• **YES** - Specify the role(s) played.<br><br>* typeset	(ARCWEST IA# M12PG00021); grant from the Office of Naval Research, Marine Mammals and Biology Program (Award Number: N000141812792); internal-NOAA grant from the NOAA Fisheries, Office of Science and Technology Ocean Acoustics Program.<br><br>JC - ASAMM visual data – Interagency Agreements between BOEM and AFSC including M07RG13260, M11PG00033, M16PG00013, and M17PG00031<br><br>KS -Marine Mammal DBO Watch visual data: National Science Foundation awards ARC-1107106) ARC-0855828, PLR-1603259, NPRB A94-00, ONR N00014-17-1-2274<br><br>JG - Benthic infauna Shipboard sample collections and post-cruises analyses: to J. Grebmeier and L. Cooper from the NSF Arctic Observing Network (AON) program (1204082, 1702456 and 1917469) and the NOAA Arctic Research Program (CINAR 22309.07 and 25984.02). Manuscript preparation to JMG provided by NSF AON grant #1917469.<br><br>SM & SO – no funding received for this study
**Competing Interests**<br><br>Use the instructions below to enter a competing interest statement for this submission. On behalf of all authors, disclose any competing interests that could be perceived to bias this work—acknowledging all financial support and any other relevant financial or non-financial competing interests.<br><br>This statement is required for submission and **will appear in the published article** if the submission is accepted. Please make	The authors have declared that no competing interests exist

Powered by Editorial Manager® and ProduXion Manager® from Aries Systems Corporation

sure it is accurate and that any funding sources listed in your Funding Information later in the submission form are also declared in your Financial Disclosure statement.<br><br>View published research articles from *PLOS ONE* for specific examples.<br><br>**NO authors have competing interests**<br><br>Enter: *The authors have declared that no competing interests exist.*<br><br>**Authors with competing interests**<br><br>Enter competing interest details beginning with this statement:<br><br>*I have read the journal's policy and the authors of this manuscript have the following competing interests: [insert competing interests here]*<br><br>* typeset	
**Ethics Statement**<br><br>Enter an ethics statement for this submission. This statement is required if the study involved:<br><br>• Human participants<br>• Human specimens or tissue<br>• Vertebrate animals or cephalopods<br>• Vertebrate embryos or tissues<br>• Field research<br><br>Write "N/A" if the submission does not require an ethics statement.<br><br>General guidance is provided below. Consult the submission guidelines for detailed instructions. **Make sure that all information entered here is included in the Methods section of the manuscript.**	NOAA, Alaska Fisheries Science Center, Marine Mammal Research Permit for Acoustic and Visual data<br><br>NSF Arctic Observing Network (AON) program (1204082, 1702456 and 1917469) and the NOAA Arctic Research Program (CINAR 22309.07 and 25984.02) for benthic data

**Format for specific study types**<br><br>**Human Subject Research (involving human participants and/or tissue)**<br>• Give the name of the institutional review board or ethics committee that approved the study<br>• Include the approval number and/or a statement indicating approval of this research<br>• Indicate the form of consent obtained (written/oral) or the reason that consent was not obtained (e.g. the data were analyzed anonymously)<br><br>**Animal Research (involving vertebrate animals, embryos or tissues)**<br>• Provide the name of the Institutional Animal Care and Use Committee (IACUC) or other relevant ethics board that reviewed the study protocol, and indicate whether they approved this research or granted a formal waiver of ethical approval<br>• Include an approval number if one was obtained<br>• If the study involved *non-human primates*, add *additional details* about animal welfare and steps taken to ameliorate suffering<br>• If anesthesia, euthanasia, or any kind of animal sacrifice is part of the study, include briefly which substances and/or methods were applied<br><br>**Field Research**<br><br>Include the following details if this study involves the collection of plant, animal, or other materials from a natural setting:<br>• Field permit number<br>• Name of the institution or relevant body that granted permission	
**Data Availability**<br><br>Authors are required to make all data underlying the findings described fully available, without restriction, and from the time of publication. PLOS allows rare exceptions to address legal and ethical concerns. See the PLOS Data Policy and FAQ for detailed information.	Yes - all data are fully available without restriction

Powered by Editorial Manager® and ProduXion Manager® from Aries Systems Corporation

A Data Availability Statement describing where the data can be found is required at submission. Your answers to this question constitute the Data Availability Statement and **will be published in the article**, if accepted.<br><br>**Important:** Stating 'data available on request from the author' is not sufficient. If your data are only available upon request, select 'No' for the first question and explain your exceptional situation in the text box.<br><br>Do the authors confirm that all data underlying the findings described in their manuscript are fully available without restriction?	
**Describe where the data may be found in full sentences. If you are copying our sample text, replace any instances of XXX with the appropriate details.**<br><br>• If the data are **held or will be held in a public repository**, include URLs, accession numbers or DOIs. If this information will only be available after acceptance, indicate this by ticking the box below. For example: *All XXX files are available from the XXX database (accession number(s) XXX, XXX.)*.<br>• If the data are all contained **within the manuscript and/or Supporting Information files**, enter the following: *All relevant data are within the manuscript and its Supporting Information files.*<br>• If neither of these applies but you are able to provide **details of access elsewhere**, with or without limitations, please do so. For example:<br><br>*Data cannot be shared publicly because of [XXX]. Data are available from the XXX Institutional Data Access / Ethics Committee (contact via XXX) for researchers who meet the criteria for access to confidential data.*<br><br>*The data underlying the results presented in the study are available from (include the name of the third party*	Marine mammal sighting data from DBO research cruises are available here: arcticdata.io (doi:10.18739/A26T0GX06).<br>The ASAMM data are available here:<br>https://www.fisheries.noaa.gov/resource/data/1979-2019-aerial-surveys-arctic-marine-mammals-historical-database<br>PAM Data<br>Alaska Fisheries Science Center, 2021: AFSC/NMML: Acoustics long-term passive monitoring using moored autonomous recorders in the Bering, Chukchi, and Western Beaufort Seas, 2007-2012, https://www.fisheries.noaa.gov/inport/item/17343.<br><br>Crustacean abundance and coincident environmental data are available on the DBO project page at the NSF Arctic Data Center archive, here" <https://arcticdata.io/catalog/portals/DBO/Data><br><br>Note: When macrofaunal replicates were only 2-3 grabs/station (2016-2019) due to covid laboratory closures, the data sets will be available on the authors website until full processing of all replicates occurs (https://arctic.cbl.umces.edu).<br><br>NCEP winds are available here:<br>https://psl.noaa.gov/data/gridded/data.ncep.reanalysis.html<br><br>Bering Strait mooring data (including A3 transports) are available here:<br>http://psc.apl.washington.edu/HLD/Bstrait/Data/BeringStraitDownloadregister.html<br><br>additional information on Bering Strait moorings here:<br>http://psc.apl.washington.edu/HLD/Bstrait/bstrait.html

and contact information or URL).<br>• This text is appropriate if the data are owned by a third party and authors do not have permission to share the data.<br><br>* typeset	
Additional data availability information:	

Manuscript
Click here to access/download;Manuscript;Manuscript GrayWhales_DBO-PLoS.rev.docx

Changes in gray whale phenology and distribution related to prey variability and ocean biophysics in the northern Bering and eastern Chukchi seas

Sue E. Moore[1*], Janet T. Clarke[2], Stephen R. Okkonen[3], Jacqueline M. Grebmeier[4], Catherine L. Berchok[5], Kathleen M. Stafford[6]

[1]Center for Ecosystem Sentinels, University of Washington, Seattle, WA 98105, USA

[2] Cooperative Institute for Climate, Ocean and Ecosystem Studies, University of Washington, Seattle WA 98105, USA

[3]Institute of Marine Science, University of Alaska Fairbanks, Fairbanks, Alaska 99775, USA

[4]Chesapeake Biological Laboratory, University of Maryland Center for Environmental Science, Solomons, MD 20688, USA

[5]Marine Mammal Laboratory, Alaska Fisheries Science Center, NOAA, Seattle, WA 98115 USA

[6]Applied Physics Laboratory, University of Washington, Seattle, WA 98105, USA

*Corresponding author: moore4@uw.edu

ABSTRACT

Changes in gray whale (*Eschrichtius robustus*) phenology and distribution are related to observed and hypothesized prey availability, bottom water temperature, salinity, sea ice persistence, integrated water column and sediment chlorophyll *a*, and patterns of wind-driven biophysical forcing in the northern Bering and eastern Chukchi Seas. This portion of the Pacific Arctic includes four Distributed Biological Observatory (DBO) sampling regions. In the Bering Strait area, passive acoustic data show gray whale phenology changed coincident with unprecedented wintertime sea ice loss there in 2017-2019, although some whales were seen in on DBO cruises. In the northern Bering Sea, changes in gray whale distribution indicate that

they continue to target a shrinking field of infaunal amphipods, with a significant decrease in prey abundance ($r = -0.313$, $p<0.05$) observed in the DBO 2 region over the 2010-2019 period. In the eastern Chukchi Sea, sightings during broad scale aerial surveys show that gray whale distribution is associated with localized areas of high infaunal crustacean abundance. Although abundance was unchanged in DBO regions 3, 4 and 5, a mid-decade shift in gray whale distribution suggests that targeted prey may now regularly include epi-benthic and pelagic species. Specifically, in the northeastern Chukchi Sea, increased sighting rates (SR = whales/km) associated with an offshore shift in distribution was positively correlated with large scale and local wind patterns conducive to increased availability of krill there. In the southern Chukchi Sea, gray whale distribution clustered in all years near an amphipod-krill 'hotspot' associated with a 50-60m deep trough. We discuss potential impacts of observed and inferred prey shifts on gray whale nutrition in the context of an ongoing unusual gray whale mortality event. To conclude, we use the conceptual Arctic Marine Pulses (AMP) model to frame hypotheses that may guide future research on whales in the Pacific Arctic marine ecosystem.

Introduction

Arctic and sub-arctic marine ecosystems are changing much faster than predicted [1]. Since the advent of satellite records in 1979, sea-ice areal extent has diminished by about 50% at the September minimum, with a roughly 75% year-round reduction in thickness of multi-year ice. This fundamental shift has not been linear in the Pacific Arctic region; rather, there were dramatic step-changes of sea-ice loss in late summer 2007 and 2012, and in winter 2017, 2018 and 2019 near Bering Strait [2]. Ocean temperatures have risen across the Arctic, driven both

by increased solar insolation that is no longer reflected back into the atmosphere by sea ice and by transport of warm ocean water from the south into sub-arctic and arctic regions [3]. In the Pacific Arctic region, the loss of sea ice has been accompanied by ocean warming and freshening [4, 5], with 2014-2018 marking a period of increased ocean-atmosphere heat exchange coincident with unprecedented low ice cover [6]. The combination of sea-ice loss and warmer seawater has reset the clock on ecological processes in the Pacific Arctic [7], with the Bering Strait region described as in a state of transformation [8]. Overall, primary production is initiated earlier in spring, with enormous blooms sometimes encountered under thin sea ice resulting in an overall 57% increase in net productivity [9]. Changes in primary productivity vary at regional and local scales, with production in the Chukchi Sea the highest in the Pacific Arctic region [10]. The biophysical impacts of reduced sea ice, increased ocean temperatures and primary production, combined with shifting atmospheric and ocean dynamics, can drive swift and fundamental changes near the base of marine food webs, the trophic level important to gray whales (*Eschrichtius robustus*).

Gray whales are unique among mysticete whales in that they can suction sediment from the sea floor and effectively sieve out the infaunal prey on their short, coarse baleen (Fig 1A). Although infaunal amphipods are common prey, gray whales are capable of efficiently feeding on epi-benthic prey swarms (Fig 1B), pelagic zooplankton aggregations, and even fish roe at the sea surface [11]. In the northern Bering and Chukchi Seas, feeding gray whales ~~are~~ most commonly ~~associated with~~ infaunal amphipods [12, 13], although epi-benthic and surface swarms of euphausiids (*Thysanoessa* spp.; hereafter, krill), eurisid amphipods (*Pontogeneia makarovi*), or cumaceans (*Diastylus glabra*) are sometimes the targeted prey [14,15]. Reports

on fine-scale feeding behavior at coastal study sites ~~along their migration route~~ describe the ease with which gray whales can switch between various prey species based upon availability and sometimes size [16, 17]. While apex predators, such as marine mammals and birds, are now commonly recognized as ecosystem sentinels [18], gray whales were one of the first cetacean species so described. Specifically, evidence of connections between changes in gray whale migration timing and distribution with shifts in their environment were summarized for six environmental factors, including oceanographic indices (i.e., Pacific Decadal Oscillation and El Nino Southern Oscillation), sea ice loss (N Bering) and thinning (W Beaufort), and shifts in infaunal and epi-benthic prey availability [19]. Taken together, these observations made a compelling case for gray whales as effective sentinels of ecosystem alteration in North Pacific and western Arctic ecosystems.

Figure 1. Gray whale feeding on infaunal amphipods, resulting in mud plumes (A), and a gray whale skim feeding on krill east of Pt. Barrow, Alaska (B).

The Distributed Biological Observatory (DBO) was initiated in 2010 to provide standardized sampling to investigate biological responses to the rapid physical changes ongoing in the Pacific Arctic marine ecosystem (Fig 2A) [20]. An opportunistic marine mammal watch was included in the standard DBO protocol, to leverage the capacity of marine mammal and other upper trophic level (UTL) species to act as sentinels of ecosystem variability and reorganization [21, 22]. Like fishes and seabirds, marine mammals rely on finding dense aggregations of prey to forage successfully. As a result, shifts in their ecology (i.e., phenology, distribution, and abundance) can signal changes in marine ecosystem trophic structure, which are in turn reflected physiologically by changes in diet and body condition [23] In addition to sightings during DBO cruises, a robust program of marine mammal research has been

conducted in the Pacific Arctic region, comprised of year-round Passive Acoustic Monitoring (PAM) of species-specific calls [24] and seasonal broad-scale Aerial Surveys of Arctic Marine Mammals (ASAMM) [25]. Changes in baleen whale phenology and seasonal distribution have been described based upon some of these data, with correlations to biophysical processes [26, 27] and details on prey availability included when possible [13, 28].

Figure 2. The Distributed Biological Observatory (DBO) in the Pacific Arctic (A), and a schematic of the conceptual Arctic Marine Pulses model (B) depicting links among biophysical aspects of the Pacific Arctic marine ecosystem.

Here, we identify changes in gray whale phenology and seasonal distribution, based upon a compilation of information from the DBO, PAM, and ASAMM programs. We then relate those shifts to changes in ocean biophysics that likely impact availability of their prey, including pelagic-benthic coupling, advection, ocean warming and freshening, and large and local-scale wind forcing. The conceptual Arctic Marine Pulses (AMP) model combines these biophysical factors into a regional framework (Fig 2B) [29], which we use to contrast the advection and pelagic-benthic coupling drivers active in the Bering Strait region (DBO regions 2 & 3) to those factors combined with seasonal sea ice retention and wind-forcing dynamics in the northeastern Chukchi Sea (DBO regions 4 & 5). Notably, the inclusion of long-term measures of infaunal crustacean abundance and species composition in DBO regions 2-5 provides direct evidence of, and fosters insights into, the impacts of shifting ocean biophysics on gray whale prey. The potential effects of observed and inferred prey alteration on gray whale nutrition are discussed in the context of an unusual mortality event that began in 2019 resulting in *ca.* 10-fold increase in annual number of stranded whales [30]. We close with suggested hypotheses that might guide future research on the ecology of gray and other baleen whales that may

enhance their capacity to act as sentinels of ongoing transformation of the Pacific Arctic marine ecosystem.

Methods

Gray whale passive acoustic and visual sampling

Our assessment of gray whale phenology, in this case defined as timing of arrival and departure from the Bering Strait region, is based on detection of their distinctive knock-like 'bongo' calls or 'Class 1 calls' [31]. Gray whale moans or 'Class 3 calls' [31] were also included when detected with 'bongo' calls and when there were no accompanying humpback sounds with which they could be confused. Data were recorded on instruments deployed in or near DBO regions 2 and 3 from 2012-2019 as part of an extensive marine mammal PAM program [24]. Instruments were set to record on a duty cycle of 30% to extend battery life for a full year, with a sample rate of 16384 Hz for an effective bandwidth of 10-8192 Hz, which is sufficient to record all known gray whale signals [31]. Daily call detections were binned in 10-minute increments and normalized by recording effort, resulting in call histograms which depict the percent of calling activity/day, rather than an actual count of calls recorded. We examined histograms of gray whale calling activity at each site to identify ~~phenological changes in annual pattern~~.

Our summary of gray whale summer-autumn distribution is based on sightings made during marine mammal watches on 22 DBO cruises (Table S-1), and on sightings made during the broad scale ASAMM program for the period 2009-2019 [25]. On DBO cruises, a visual watch for marine mammals was conducted during daylight hours when the ship was transiting between sampling or mooring stations augmented by scans around the ship each hour when

the ship was on station. An observer trained in marine mammal species identification used naked eye and handheld binoculars to scan a 120° arc forward of the ship (abeam, to +30° of the bow) out to the horizon. When two people were available to stand watch, the full 180° arc forward of the ship was scanned to the horizon. The watch stander was often assisted by other scientific party personnel and the ship's cr[illegible]. The watch was curtailed when sea state exceeded Beaufort 05 (wind speed ~25kts), or visibility was reduced to < 1km by precipitation or fog

During the ASAMM program, which sampled an area inclusive of DBO 3-5, line-transect aerial surveys were conducted from July-September using twin engine aircraft outfitted with left- and right-side bubble windows. Dedicated observers stationed at each window reported all marine mammal sightings and associated environmental conditions to a data recorder. Sightings of large whales were briefly circled over to confirm species identity, group size, behavior, and presence of calves. Surveys were conducted whenever weather conditions allowed (i.e., sea state ≤Beaufort 05; cloud ceiling consistently >335 m). Offshore transects were oriented perpendicular to the coastline to allow sampling across isobaths and prevailing currents, and to best assess marine mammal density gradients; a coastal transect was flown *ca.* 1-km offshore to better document nearshore habitat. Only sightings made during transect survey effort were included in the analyses presented here.

To examine changes in gray whale distribution in the context of environmental variability, ASAMM data were divided into two areas for analysis: (1) the southern Chukchi Sea, referred to as Hope Basin area and inclusive of DBO 3; and (2) the northeastern Chukchi Sea, referred to as the Wainwright and Peard Bay areas, inclusive of DBO regions 4 & 5. Gray whale

sighting rates (SR) were calculated for each area as the number of whales seen/offshore transect kilometer by month (July, August, September) and year (Table S-2). Effort and sightings on the coastal transect were not included in SR analyses to avoid bias towards nearshore areas.

Gray whale prey and environmental variability

Environmental factors relevant to the influence of pelagic-benthic coupling on gray whale infaunal prey were evaluated based upon a multi-decadal record of macrobenthos and sediment dynamics [32], with special focus on stations dominated by crustacean species at the start of the 2010-2019 time series [33]. Gray whale crustacean prey were analyzed with attention to changes in abundance and species composition in response to trends in sea ice loss, ocean temperature, salinity, and measures of chlorophyll a both in the water column and in surface sediment. While infaunal crustacean data from 2010-2015 are fully processed, those for 2016-2019 represent only 2-3 grabs/station due to laboratory closures related to the Covid-19 pandemic. In this case, we used the average amphipod abundance in the statistical analysis, although all environmental data are final. Note that for the DBO 4 site stations extended along 3 transect lines in the bounding box and data from all stations where crustaceans were dominant at the start of the time series were included in the analysis for that region. Statistical analyses comparing crustacean infauna abundance and environmental data were completed using the statistical software JMP™ Pro 15.20 (SAS Institute Inc., SAS Campus Drive, Cary, NC, USA 27513).

Environmental factors potentially indicative of the influence of advection and circulation on gray whale epi-benthic and pelagic prey availability were evaluated through application of an iterative correlation analysis (ICA) method [34,35]. The ICA method is a simple supervised

machine learning tool that uses, in the present case, a multiyear time series of gray whale SR in the Hope Basin, Wainwright, or Peard Bay areas as a training set to identify a statistically similar interannual time series of a seasonally-averaged environmental factor (i.e. volume, heat, freshwater transports) measured at the ‘climate mooring’ north of Bering Strait [4; mooring A3], or local- an[illegible]rge-scale wind regimes derived from National Centers for Environmental Prediction/National Center for Atmospheric Research daily sea level wind products [36]. For an environmental factor with time series information at a single location (e.g. transport at mooring A3), ICA identifies the seasonal start and end dates for which correlations between seasonally-averaged transports and gray whale SRs are statistically significant. For an environmental factor with time series information at multiple locations (e.g. NCEP winds), ICA identifies the seasonal start and end dates that maximize the ocean area north of the Bering Sea shelf break over which correlations between seasonally-averaged winds and gray whale SRs are statistically significant. Results for both single and multiple location ICA are summarized in the form of a heat map matrix that identifies the best-fit start and end dates from a range of candidate seasonal averaging periods.

Results

Changes in gray whale phenology and distribution

Acoustic detections

A change in gray whale acoustic detections was observed in the northern Bering Sea (DBO 2) where the period of calling activity extended from late May through November in 2012-2015, shortened t[illegible]une to mid-September in 2016, and was followed by a near cessation of call detections in 2017-2019 (Fig 3A). In the southern Chukchi Sea (DBO 3), consistent calling

activity extended from mid-June through October in 2012-2016. In 2017 call detections ended a month earlier (September), and there was a near cessation of calling activity throughout 2018-2019 (Fig 3B). These changes in calling activity suggest that gray whales departed DBO regions 2 and 3 earlier each year after 2016 and 2017, respectively.

Figure 3. Gray whale annual calling activity in the northern Bering (DBO 2) and southern Chukchi (DBO 3) Seas, 2012-2019. Red arrows denote dramatic drop-off in calls coincident with 2017-2019 winter sea ice loss event.

Visual detections

Gray whales were seen during marine mammal watches on DBO cruises in all years (Fig 4A). Areas of concentration included the northern Bering and southern Chukchi Seas, and waters offshore Wainwright and along the coast between Pt. Franklin and Pt. Barrow. The timing and track of DBO cruises varied among years, limiting inferences that can be drawn from these data. Of note, however, is that gray whales were consistently seen at the western end of the DBO 3 transect line and west of the International Date Line in years that ships had access to those waters (2009, 2010, 2012); and, in contrast to the PAM data, whales were seen in DBO regions 2 and 3 in 2017-2019 after the aforementioned loss of winter sea ice in those areas.

Figure 4. Gray whale distribution from sightings made during marine mammal watches on DBO cruises (A), and the broad scale Aerial Surveys of Arctic Marine Mammals program (B).

The ASAMM data provide a more robust accounting of gray whale distribution relative to survey effort north of 67°N in the Chukchi Sea. Over the eleven-year study period, there were 1,333 sightings of 2,358 gray whales from July-September, with most whales seen in south-central and northeastern waters (Fig 4B; Table 1) similar to findings from the DBO cruise

data. Interannual variability in SR for the entire study area ranged from a high of 2 whales/100km in 2019 to a low of 0.5 whales/100km in 2010. In 2015, there was a *ca.* 110 km (60 nm) shift in gray whale distribution away from the coastal habitat between Pt. Franklin and Pt. Barrow to waters offshore Wainwright and near the head of Barrow Canyon. In the south-central Chukchi Sea, gray whale distribution clustered in a benthic trough 'hotspot' in all years, with sightings extended further south after 2015. Since gray whales spend much of their time feeding while in the Chukchi Sea, these changes in distribution were examined (i) with reference to observed changes in infaunal crustacean prey abundance and community composition, and (ii) by correlation of sighting rates with environmental factors associated with availability of epi-benthic and pelagic prey, as described in the next two sections.

Table 1. Annual ASAMM survey effort (km), number of gray whale sightings, number of whales seen and sighting rate (SR=whale/km), July-September, 2009-2019. Includes all sightings and effort on transect, including coastal transects.

**Year**	**Effort km**	**No. sightings**	**No. gray whales**	**SR**
2009	15,560	88	115	0.0074
2010	16,089	52	74	0.0046
2011	20,010	102	139	0.0069
2012	21,077	124	221	0.0105
2013	21,768	67	110	0.0051
2014	19,032	164	291	0.0153
2015	20,865	98	185	0.0089
2016	21,381	161	366	0.0171
2017	23,021	207	384	0.0167
2018	15,589	167	311	0.0199
2019	13,121	103	162	0.0123
Total	207,513	1,333	2,358	0.0114

Changes to infaunal crustacean abundance, community composition, and associated environmental factors

Our multi-decadal record of infaunal crustacean abundance provided the means to directly track changes to gray whale benthic prey in the productivity hotspots of DBO regions 2-5 (Fig 5). In the northern Bering Sea, the clearest change during the 2010-2019 period was a significant decrease (r=-0.31[illegible]≤0.05) in amphipod abundance in the DBO2 region (Table 2). Especially notable was the decline in crustacean abundance at the northwest sampling sites starting in 2015 (Fig 6A: open square symbol). Note that the northwest site (U[illegible]4) was the only station in the DBO2 region that showed a significant decline from 1999-2015 [32], thus the decline through 2019 confirms a continued faunal shift for gray whale amphipod prey in this region.

Figure 5. Abundance of infaunal crustaceans predi[illegible]d from DBO sampling stations in the northern Bering and eastern Chukchi Seas, 2009-2019. Note that the DBO4 stations evaluated were inclusive of three transects within a DBO4 bounding box that are considered replicates for this study. Only stations dominated by crustaceans by abundance (yellow-red color pattern) were used in the analysis.

Figure 6. Measurements of dominant infaunal crustacean abundance in DBO regions 2-5. Stations used were those where crustaceans were dominant by abundance at the start of the time series. The time series crustacean abundance areas are within DBO 2 (all stations), DBO 3 (inclusive of stations S to N in the DBO3 hotspot bounding box and the western end of the DBO3 E to W transect line), DBO 4 (multiple stations within the DBO4 bounding box of high abundance stations), and DBO 5 (high dominant crustacean abundance areas in the central and eastern stations on this line). Note that DBO 4 became a time series site starting in 2013, and that replicate data are not currently available for all relevant stations in DBO4 and 5 due to lab closure due to the Covid pandemic during 2020. Key for [illegible]mbols: closed symbol are the southern time series stations in the DBO bounding box, open symbol are the northern time series stations in each individual DBO2-5 region.

Table 2. Changes in infaunal crustacean community composition and abundance, bottom water temperature and salinity, sea ice persistence (days from Sept-to Sept), and integrated water column and

sediment chlorophyll in DBO regions 2-5 for the period 2010-2019. Values are average ± s.d. Key: **bold** is significant increase (+) or decrease (-) over the decade, ***=p<0.05**, ****=p<0.01**; ns=not significant; (#)= number of stations, with sea ice persistence number of years. Dominant species/family: Ampeliscidae (*Ampelisca macrocephala*, *A. birulai*, *Byblis* sp.), Isaeidae (*Protomedeia fasciata, Photis* sp.), Leuconidae (*Eudorella pacifica),* Phoxocephalidae (*Grandifoxus* sp., *Heterophoxus* sp.), Pontoporeidae (*Pontoporeia femorata*).

	**REGION**			
**Parameters**	**DBO2**	**DBO3**	**DBO4**	[illegible]
	2010-2019	2010-2019	2010-2019	
Dominant crustacean families (by abundance)	Ampeliscidae, Isaeidae	Ampeliscidae, Isaeidae, Pontoporeidae	Isaeidae, Phoxocephalidae, Pontoporeidae	Ph[illegible]
Abundance (no/m2)	5553 ± 6609<br>**r= -0.313* (**51)	3839± 5469<br>r= 0.039, ns (78)	3870 ± 5485<br>r= 0.216, ns, (30)	
Bottom water temperature (°C)	1.98 ± 1.20<br>**r= +0.678**** (51)	2.74 1.69<br>**r = +0.378**** (91)	-0.35 ± 1.52<br>**r = +0.798**** (40)	
Bottom water salinity (psu)	32.56 ± 0.31<br>**r= +0.367*** (51)	32.47 ± 0.39<br>**r= +0.245*** (91)	32.45 ± 0.30<br>**r= -0.739**** (40)	
Sea ice persistence (days/year)	143 ± 28<br>**r= -0.756**** (10)	177 ± 17<br>**r= -0.731**** (10)	222 ± 35<br>**r = -0.926**** (5)	
Integrated chlorophyll *a* (mg/m2)	66.73 ± 53.43<br>**r= +0.370*** (51)	222.84 ± 200.40<br>**r = +0.316*** (87)	117.70 ± 102.02<br>**r = -0.394*** (28)	
Sediment chlorophyll *a* (mg/m2)	19.54 ± 7.24<br>**r= +0.399*** (51)	20.80 ± 6.81<br>r = 0.034, ns (91)	14.04 ± 8.09<br>**r = +0.477*** (40)	

In the southern Chukchi Sea, comparatively small-bodied amphipod species from three families were found where gray whales were seen feeding in the DBO 3 region (Fig 5; Table 2). There was no significant trend in infaunal crustacean abundance in this region, with the highest crustacean abundance consistently observed in the hotspot sampled by the northern stations (Fig 6B: open symbol). Notably, although abundance remained stable over time, there was a shift in community composition, whereby the northernmost stations of the DBO 3 hotspot are now dominated by *Pontopoporeia femorata* (F. Pontoporeidae), which replaced the *Byblis* sp. (F. Ampelicidae) in the mid-2000s and has remained the dominant crustacean species since that time; meanwhile the small F. Isaeidae amphipods have remained second in abundance throughout the last decade.

In the northeastern Chukchi Sea, feeding gray whales were seen where both comparatively large infaunal amphipods (F. Pontoporeidae) and a variety of small infaunal crustacean species (F. Isaidae., F. Phoxochelidae) were found in a localized area along the eastern flank of Hanna Shoal in DBO 4. The infauna at all 6 stations in the time series were equitable in abundance, but there was notably higher amphipod abundance in 2017 at the nearshore stations (Fig 6C: solid symbol) in a region close to the head of Barrow Canyon. In DBO 5, both the larger amphipods (F. Ampeliscidae) and smaller amphipods (F. Isaeidae and F. Phoxocephalidae) and cumaceans (F. Leuconidae*)* were abundant along the narrow shelf nearshore (Fig 5[illegible] Table 2). Although there was no significant trend in infaunal crustacean abundance in the DBO 5 region, there was an indication of a possible decline starting in 2017 (Fig 6D). Unfortunately, sea ice cover in 2018 precluded sampling the DBO5 stations and sorting of 2019 samples is in progress.

A summary of changes in environmental factors associated with the sampling of infaunal crustaceans provides context, but no simple answers as to why there was a significant change in abundance only in the DBO 2 region (Table 2). Sea ice persistence has *decreased* and integrated water column chlorophyll has *increased* in all DBO regions 2-5, suggesting an enhanced pelagic food supply throughout the region north of St. Lawrence Island. Notably, sediment chlorophyll increased only in DBO 2 and DBO 4. This suggests food supply to infaunal crustaceans has increased in those regions, making the *decline* of amphipods in DBO 2 unexpected and indicating that some combination of environmental factors is driving abundance. In reviewing those factors, we note that bottom water temperature has increased in all regions except DBO 5, accompanied by increased bottom water salinity in DBO 2 and DBO 3 (Table 2). Bottom water became significantly fresher in DBO 4, likely due to increasing sea ice melt and perhaps freshwater pulses from the south. There was no change in bottom water salinity in DBO 5, likely due to the seasonal complexity of Barrow Canyon ocean dynamics.

Environmental factors related to gray whale epi-benthic and pelagic prey availability

Since gray whales also feed on epi-benthic and pelagic prey, correlational analyses were used to investigate drivers of Chukchi Sea circulation (regional wind forcing and transport through Bering Strait) that potentially alter availability of these prey and thereby result in changes in gray whale distribution and sighting rates (SR) in the Chukchi Sea. In the northeastern Chukchi Sea, interannual changes in gray whale monthly distribution and SR depict a shift in foraging away from the nearshore Peard Bay area (2009-2014) to waters

offshore Wainwright (2015-2019), near the head of Barrow Canyon (Fig 7). The shift in distribution and SR is most evident for the month of [illegible]uly, with a near-abandonment of the Peard Bay area in August and September. To investigate this shift, averaged July-August-September (JAS) SRs for Wainwright (W) and Peard Bay (PB; Fig 8A) were used to create a 2009-2019 time series of fractional SR, ($W_F = W_{JAS}/(W_{JAS} + PB_{JAS})$). This served as the ICA training set (Fig 8B) to identify 2009-2019 time series of seasonally-averaged winds within the Bering-Chukchi-Beaufort domain that were significantly correlated ($r > 0.60$, $p<0.05$, 9 d.f.) with the fractional SR. The ICA heatmap shows three loci, all with start dates in early June (Fig 8C), with averaging periods of 21 days (end date late June; best fit indicated by diamond symbol), ~45 days (end date late July) and ~100 days (end date mid-September). The resulting wind regimes (similar for all three loci in that the action center is the region north of ~70° N and in which statistically-significant variability primarily occurs with the E-W component of the wind field; blue contours) suggest JAS foraging conditions are relatively better in the Peard Bay area when late-spring and summer winds are easterly and persistent (warm color shading) over the northern Chukchi and southern Beaufort (Fig 8D). Conversely, gray whale foraging on epi-benthic or pelagic prey (e.g. krill) is likely better in the Wainwright area when late-spring and summer winds are weak, variable (cool color shading), or westerly (Fig 8E).

Figure 7. Interannual variability in gray whale monthly distribution and sighting rates (SR) for Wainwright and Peard Bay analytical areas in the northeastern Chukchi Sea, 2009-2019.

Figure 8. Gray whale sighting rates (SR) and wind forcing, including: mean July-September SR in the Wainwright and Peard Bay analytical areas (A); Wainwright area SR fraction (B); ICA heat map showing the ocean area of the Bering-Chukchi-Beaufort domain over which correlations between the SR fraction and time-averaged winds are statistically-significant ($r > 0.602$, $p<0.05$; 9 degrees of freedom, two-tailed test) over a range of start dates and averaging periods (C); mean atmospheric circulation from 5 June to 25 June for years in which the Wainwright SR fraction was less than average (i.e., the Peard Bay SR fraction was greater

than average) (D); mean atmospheric circulation from 5 June to 25 June for years in which the Wainwright SR fraction was greater than average (i.e., the Peard Bay SR fraction was less than average) (E). Mean wind vectors plotted at every 2nd ith grid point. Correlation contours at r=0.60 ($p < 0.05$) and 0.74 ($p < 0.01$).

Interannual variability in gray whale monthly distribution and SR in the Hope Basin area depicts a more stable situation, with no clear relationship with local or regional winds. Sightings were clustered around a quasi-stationary front associated with a bathymetric trough southwest of Pt. Hope in all years 2014-2019, with a southern extension of sightings evident in the combined July distribution (Fig 9). Except for 2015, the annual SR for gray whales in the Hope Basin area was consistently higher from 2014-2019 than for the years 2009-2013, with no clear relationship with local or regional winds (Fig 10). Combined monthly SR showed intra and interannual variability, with peaks in July (2017 and 2019), August (2014 and 2017), and September (2014 and 2016).

Figure 9. Interannual variability in gray whale monthly distribution and sighting rates (SR) for the Hope Basin area in the southern Chukchi Sea, 2014-2019.

Figure 10. Gray whale combined July, August September sighting rates (SR) in the Hope Basin area in the southern Chukchi Sea, 2009-2019.

Interannual variations in gray whale SR in both the southern and northeastern Chukchi Sea were used as ICA training sets to identify time series of seasonally-averaged transports at the A3 ‘climate’ mooring site north of Bering Strait. ICA results indicate that winter-to-early-spring volume, heat, and freshwater transports are positively correlated with the combined JAS SR in the Wainwright area and negatively correlated with combined JAS SR in the Peard Bay area (Table 3). In the southern Chukchi Sea, interannual variations in August and September SR were negatively correlated with volume, heat, and freshwater transports.

Table 3. Coefficients of linear correlation between time-averaged transport at mooring A3 (Woodgate 2018) and gray whale sighting rates (SR). Correlations in non-bold typeface are significant at $p < 0.05$. Correlations in **bold** are significant at $p < 0.01$; 4df, $p<0.05$, $|r| > 0.811$; $p<0.01$, $|r| > 0.917$; 9df, $p<0.05$, $|r| > 0.602$; $p<0.01$, $|r| > 0.735$. JAS = July-August-September.

	Volume transport	Heat transport	Freshwater transport
**Wainwright JAS SR** (9df)	**0.799**, 3-month avg Feb GW year – Apr GW year	**0.932**, 3-month avg Feb GW year – Apr GW year	**0.829**, 3-month avg Feb GW year – Apr GW year
**Peard Bay JAS SR** (9df)	Not significant	**-0.766**, 1-month avg Jan GW year	-0.683, 4-month avg Jan GW year – Apr GW year
**Hope Basin Aug SR** (4df)	-0.880, 1-month avg Feb GW year	-0.866, 4-month avg Jun prior year – Sep prior year	-0.871, 1-month avg Feb GW year
**Hope Basin Sep SR** (4df)	**-0.962**, 11-month avg Sep prior year – Jul GW year	**-0.994**, 10-month avg Nov prior year – Aug GW year	**-0.991**, 12-month avg Aug prior year – Jul GW year

A summary of results relating variability in gray whale sighting rates (SR) to circulation drivers (A3 transport and winds), bathymetry, and likely whale prey highlights contrasts among the three analytical areas in the Chukchi Sea (Table 4). Notably, transport at A[illegible]s correlated with gray whale SR in each of the regions, while a contrasting pattern of high and low SR can be correlated with alternate wind patterns only in the Peard Bay and Wainwright areas. In sum, bathymetry and transport appear to be drivers of gray whale pelagic prey in the Hope Basin area (inclusive of the DBO 3 region), while bathymetry, transport and wind forcing play a role in the northeastern Chukchi Sea (inclusive of DBO 4 and 5).

Table 4. Summ[illegible] of bathymetry, gray whale prey and circulation drivers (transport and winds) used in correlat[illegible] analyses with gray whale (GW) sighting rates (SR) for three analytical areas in the Chukchi Sea. JAS = combined July, August and September SR. combined; N/A = not applicable.

**Analytical Area**	**Bathymetry**	**Gray whale prey** (see Table 2)	**Transport @ A3** (see Table 3)	**Winds over northern Chukchi Sea** (see Figs. 7, 8)
**Peard Bay**	Coastal shelf (20-40m deep)<br><br>GW distributed along shore from Peard Bay to Point Barrow (Fig. 5B)	Benthic species: amphipods cumaceans	**JAS GW SR** negatively correlated with average winter transports	**JAS GW SR High 2009-2014**<br><br>Strong & persistent ENE winds in northern Chukchi in summer interrupt delivery of krill to head of Barrow Canyon
**Wainwright**	Offshore shelf (40-50m deep)<br><br>GW distribution clustered at the head of Barrow Canyon, NW of Wainwright (Fig. 5B)	Benthic species: amphipods, east flank of Hanna Shoal<br><br>Pelagic & epi-benthic species: krill, possibly other spp.	**JAS GW SR** positively correlated with average winter transports	**JAS GW SR High 2015-2019**<br><br>Weak & variable winds in the northern Chukchi in summer promote the convergent delivery of krill to the head of Barrow Canyon
**Hope Basin**	Inflow shelf (~40m deep)<br><br>GW distribution localized over 50-60m trough SW of Point Hope; southward extension along central channel in 2014, 2018, 2017 (Fig. 5B)	Benthic species: amphipods, in trough 'hotspot'<br><br>Pelagic & epi-benthic species: krill, at frontal system coincident with trough slope (Bluhm et al. 2007)	**August GW SR** negatively correlated with winter or preceding summer transports<br><br>**September GW SR** negatively correlated with long-term average transports	N/A

Discussion

Gray whale ecology in the northern Bering and Chukchi Seas has been the focus of intermittent research since the late 1960s, which provides a foundation upon which to interpret the changes in their phenology and distribution reported here. For example, maps from Russian surveys from 1968-1982 show a near-continuous gray whale distribution along the

Chukotka peninsula, with clustered aggregations of hundreds of whales in the coastal and central southwestern Chukchi Sea [37]. Likewise, in a review of Russian research associated with commercial whaling, gray whales were reported as commonly seen feeding on benthic and epibenthic organisms in shallow waters along the Chu[illegible]ka peninsula where prey concentrations were greater than 100 g/m^2, with distribution extending northwestward to the Eastern Siberian Sea and a very large aggregation (~2000 whales) encountered roughly 160 km offshore between 67°40-68°15’N and 169°40’-172°W during a 1980/81 expedition [12]. Stomach contents from whales taken in Mechigmensky Bay in 1980/81 contained roughly 122 benthic species [38], with amphipods the most common; e.g. *Pontoporeia femorata* (57.9%), *Ampelisca macrocephala* (53.9%), *Byblus longicornis* (51.3%) and *Lembos arcticus* (47.3%) Similarly, a satellite tracking study of 9 gray whales offshore Chukotka in late summer 2006 found that whales remained in shallow (< 30m) nearshore waters (< 5 km from coast) and were likely feeding on dense aggregations of amphipods [39].

While maps from 1975-1980 surveys offshore Alaska depict comparatively few gray whale sightings in the southeastern Chukchi Sea, there is a distinct distribution cluster in what is now called the DBO 3 ‘hotspot’ area [40]. Conversely, the distribution of gray whales in the northern Bering Sea was extensive, with a dense cluster of sightings extending north from St. Lawrence Island through Bering Strait. This dense summertime distribution of gray whales in the DBO 2 area continued at least through the mid-1980s [41, 42], but by the early 2000s gray whale sightings were comparatively sparse and shifted northward following a shrinking pattern of amphipod distribution [43, 44]. In the northeastern Chukchi Sea, focal areas for gray whale distribution in the 1980s included coastal waters between Icy Cape and Pt. Barrow, with a

cluster of sightings near Hanna Shoal northwest of Wainwright [41]. There are no year-round acoustic data for the Bering Strait region in 1980s, but gray whales were reported in the northern Bering and Chukchi Seas from May through at least mid-October, with some whales seen as late as December seemingly following the seasonal cycle of sea ice retreat and formation [12, 40].

Changes in gray whale phenology

Gray whale phenology, as described by patterns of calling activity in the Bering Strait region, suggests little change in May-June arrival times from 2012-2016 in the northern Bering Sea (DBO 2), and through 2017 in the southern Chukchi Sea (DBO 3). Departure dates inferred from a drop in calling activity changed dramatically thereafter, shifting from November to mid-September in 2016 (DBO 2) and from October to September in 2017 (DBO 3). This shift towards earlier departure dates and shorter residency periods culminated in a near-absence of calling activity in years 2017-2019 (DBO 2) and in 2018-2019 (DBO 3). This drop in calling activity in each region after 2017 and 2018 suggests that gray whales may have abandoned formerly prime foraging areas coincident with the anomalous 2017-2019 winter sea ice loss event [45]. Alternatively, because gray whale acoustic activity was based on detection of only two call types [31, Class 1 and 3], the drop in call detections could reflect changes in age-class composition or behaviors associated with those signals, rather than whale departure [24].

Sightings of gray whales in the Bering Strait region during DBO cruises and the ASAMM surveys suggest that the abrupt changes in calling activity may indicate a modest (*ca.* 30-40 km) shift in distribution away from the recorder locations, rather than a substantial change in gray whale phenology. While propagation modeling has not been conducted at this site, evidence

from modeling studies elsewhere in the northern Bering Sea suggests that gray whale calls are not likely detected beyond a radius of about 35km (20nm). So, for example, a shift of whales northward to the remaining amphipod beds in DBO2, or westward toward the DBO 3 hotspot, could take them out of acoustic detection range of the recorder. However, because the clear truncation in gray whale calling activity coincided with anomalous wintertime sea ice loss event in 2017-2019, this potential shift in phenology and/or distribution is noteworthy. Future deployments of recorders near the remnant amphipod beds in DBO2, or the benthic trough hotspot in DBO3 could help resolve some of the uncertainty regarding shifts in gray whale phenology in the Bering Strait region.

Gray whale infaunal prey abundance, community composition, and environmental factors

Since 2010, the abundance of infaunal crustaceans that gray whales feed upon has significantly declined only in the DBO 2 region. This decline has been ongoing since the late 1980s [41, 42] leaving only the northern portion of the Chirikov Basin suitable for gray whale foraging. This change is concomitant with a shift in sediment quality due to faster currents, with the finer sediments required for tube building by *A. macrocephala* found only where current speed is reduced by land mass constriction in the offshore waters south of Bering Strait [44]. Of note, the decline of infaunal crustaceans in DBO 2 is also coincident with an increase in polychaetes and bivalves [32, 33]. Collectively, these data suggest a physiological sensitivity of the dominant amphipods to warming conditions in the DBO 2 region, with the potential to be

outcompeted by organisms more tolerant of seawater changes and able to take advantage of increasing integrated and sediment chlorophyll carbon sources.

While there was no decline in infaunal crustacean abundance in DBO 3, a shift in dominant species was observed. Specifically, *Byblis* spp. (F. Ampeliscidae) was dominant from 1984-2004, but is now largely absent there, although it continues to be abundant in the DBO 5 region. The larger amphipod *Pontoporeia femorata* (F. Pontoporeidae) is now the dominant species in the northern portion of the DBO 3 hotspot, continuing to provide a benthic prey base for gray whales in the 'hotspot' area where they also appear to feed on advected krill [14]. Infaunal crustacean abundance in DBO regions 4 and 5 remained stable and without major changes in dominant fauna, at least through 2017. Evaluation of samples currently in progress may help clarify changes in crustacean abundance at these northern DBO sites.

Environmental factors and pelagic-benthic coupling

What has influenced the changes or stability of the infaunal crustacean communities across the DBO 2-5 sites? On the face of it, the lack of change to infaunal crustacean abundance in DBO regions 3, 4 and 5 seems inconsistent with a fundamental tenant of the pelagic-benthic coupling model whereby loss of sea ice results in reduced organic carbon from ice-associated algae dropping to the sea floor and thereby cutting food supply for benthic macrofaunal communities [46]. Yet, our observations show that surface sediment chlorophyll increased as sea ice persistence decreased in DBO regions 2 and 4 (Table 2). Notably the significant decrease in sea ice persistence was associated with a significant increase in integrated water column chlorophyll in all DBO regions (Table 2). These observations support the idea that changes in the timing and magnitude of water column primary production play an

important role in carbon transfer to the seafloor [32]. Previous studies indicated that primary production is highest in the northern Bering Sea (DBO 1-2) in April/May, with a production peak north of Bering Strait (DBO 3) in June followed by peak production in the northeastern Chukchi Sea (DBO 4-5) in July [47]. Recently, however, pelagic primary production north of Bering Strait has increased in early summer [9, 48], which may be mismatched? with the arrival of zooplankton grazers thereby leaving phytoplankton production to fall to the seafloor and feed benthic macrofauna. This effect on pelagic-benthic coupling may be especially true in the DBO 3 hotspot where deposition is greatest [49].

Clearly other environmental factors, such as sea ice thinning [10], increase in ocean heat and freshwater in the Chukchi Sea [5,6], and changing sediment grain size associated with variability in current speed [32, 33, 44] are also influencing benthic macrofauna community structure. Specifically, increased water temperatures in DBO regions 2-4 is likely affecting the distribution of benthic species, as has been reported for the northeastern Atlantic where *Ampelisca* spp. have extended their distribution northward with warming seawater [50]. Of note, DBO5 stations in Barrow Canyon do not show increased bottom water temperatures, likely due to its dynamic nature and the seasonally variable water masses reported there [51]. Finally, higher salinity bottom waters seen in DBO2 and DBO3, perhaps indicative of a stronger Anadyr water signal at depth, may also influence nutrient availability to to surface waters by mixing upward of AW after storm events. By comparison, DBO4 in the NE Chukchi Sea shows a decrease in bottom water salinity and a decline in integrated chlorophyll a, likely a response to reduced brine production with declining sea ice and variable nutrient content.

Changes in gray whale distribution related to pelagic prey availability

Changes in gray whale distribution based on sightings were evident in the northeastern Chukchi Sea, and somewhat less so in the southern Chukchi Sea. Analyses of the ASAMM data show a mid-decade southwestward shift in distribution of roughly 110 km (60 nm), from coastal waters between Pt. Franklin and Pt. Barrow to waters offshore Wainwright. High gray whale sighting rates (SR) in the coastal Peard Bay area (2009-2014) were associated with strong and persistent easterly winds over the northern Beaufort-Chukchi region, while high SR in the Wainwright area (2015-2019) were associated with weak and variable winds that are conducive to krill remaining on the shelf over winter. Winter-spring transports of volume, heat, and fresh water through Bering Strait were positively correlated with gray whale SR in the Wainwright area and negatively correlated with SR in the Peard Bay area (Table 4). This lagged relationship implies an advective timescale of ~4-6 months for biophysical signals to travel from Bering Strait to Barrow Canyon, which roughly comports with reports of drifters traveling along that path in 90 days [52]. The heat signal, in particular, invites speculation that zooplankton development and survivability are enhanced in years with greater transport ultimately leading to improved summer foraging conditions near the head of Barrow Canyon. When krill remain on the northern Chukchi shelf, we postulate that they are subsequently aggregated via the convergence of northern Chukchi currents which promotes gray whale foraging near the head of Barrow Canyon. Although we have no confirmatory evidence (e.g., feces, stomach contents)

that gray whales feed on krill in this area, they have been seen skim feeding on krill in association with foraging bowhead whales east of Pt. Barrow (Fig. 1B).

Changes in gray whale distribution in the southern Chukchi Sea were evaluated analytically using ASAMM data and, for waters south of 67° N, by qualitative summary of sightings during DBO cruises. Both data sources confirmed a strong and consistent aggregation of gray whales at a prey hotspot associated with a 50-60 m trough in the central Chukchi Sea. Furthermore, sightings during DBO cruises showed that this feeding hotspot extends across the international date line, with additional sighting of gray whales common along the Chukotka coast and associated with the western boundary of the central channel.

Implications for the Gray Whale Unusual Mortality Event (UME)

In 2019, a gray whale UME was declared when the number of dead animals found stranded on beaches along their migration route between Alaska and Mexico increased roughly ten-fold [30]. The UME is officially ongoing as of the publication of this paper with the total number of dead whales totaling 216 in 2019, 172 in 2020 and 77 through 4 June 2021. A similar UME occurred in 1999-2000 and the emaciated condition of some of the dead whales during both events suggested starvation or nutritional deficit as a likely cause [30, 53]. Notably, ship strikes, entanglement in fishing gear, and predation by killer whales (*Orcinus orca*) contributed to the death toll during both events, while mortalities due to disease and biotoxins could not be clearly identified [54].

Whether or not gray whales were nutritionally stressed due to a change or reduction in prey availability related to environmental extremes, and/or to increased competition for food

resulting from their burgeoning population size, remains a pivotal question with regard to both UME events [55]. The 1999-2000 UME closely followed the powerful 1997-1998 El Niño, while the onset of the 2019 UME was subsequent to extreme ocean heat events in the North Pacific and Gulf of Alaska from 2014-2016 [56], as well as the ocean warming and wintertime sea ice loss that persisted in the northern Bering and Chukchi Seas from 2017-2019. The latter event was contributory to a mass mortality of seabirds [45] and likely influenced an ice seal UME [57].

The record on infaunal crustacean communities presented here shows that while gray whale benthic prey abundance declined in the northern Bering Sea from 2010-2019, it remained stable in the Chukchi Sea. Specifically, a significant decrease in amphipod abundance was observed in the DBO 2 region (*aka* the Chirikov Basin) of the northern Bering Sea, a decline that extends a trend that began in the mid-1980s [44]. Conversely, there was no decrease in infaunal crustacean prey abundance evident in DBO regions north of Bering Strait, although there was a shift from a smaller (*Byblis* spp) to a larger (*Pontoporeia* spp) dominant amphipod species in the DBO 3 region. Meanwhile, correlation analyses support the hypothesis that gray whales may be finding more opportunities to feed on krill near DBO regions 4 and 5 in the northeastern Chukchi Sea.

While changes in gray whale prey, either among benthic infauna or between benthic and pelagic species, will have nutritional ramifications, it seems unlikely that a change in diet alone would result in a dramatic upsurge in mortality. In other words, from the perspective of gray whale prey abundance, our observations provide context but not causation for poor nutritional condition of gray whales that feed north of Bering Strait. Conversely, behavioral

factors leading to gray whale feeding-site-fidelity in the Chirikov Basin could result in nutritional stress for a portion of the population. Indeed, there is evidence at the local scale that biophysical events that impact gray whale prey availability can lead to changes in phenology, a reduction in feeding, and signs of poor body condition. This was the case in 2005 when late spring ocean conditions off the Oregon coast markedly decreased abundance of epi-benthic mysids leaving the small population of gray whales that feed there over summer with a nutritional deficit [58].

The AMP model as a framework for future research

The Pacific Arctic marine ecosystem is in a state of transformation as it responds to amplified planetary warming [8]. In constructing the conceptual AMP model, we purposely imbedded mechanistic features such as transport through Bering Strait, pelagic-benthic coupling, advection, and upwelling as key biophysical drivers that influence biological outcomes in the region (Fig 2B) [29]. The challenge is to elucidate how these biophysical drivers interact in this productive inflow shelf system. Results from this and past studies suggests that this question may be best approached by investigating factors that (i) drive aspects of pelagic benthic coupling in the northern Bering and southern Chukchi Seas (DBO 2 and 3), and (ii) influence downstream advection, upwelling and ocean circulation dynamics in the northeastern Chukchi Sea (DBO 4 and 5). This type of approach was used to develop a mechanistic model for the persistence of benthic 'hotspots' in DBO regions 1-5, showing that those in regions 2, 3, and 5 rely on carbon supplied from upstream biological production, while those in regions 1 and 4 were supplied by local production [59]. As in past studies focused on macrofaunal biomass

[32,33], our observations of changes in infaunal crustacean prey abundance in DBO regions 2 and 3 suggest that the timing and magnitude of water column primary production, bottom water temperatures, bathymetric channeling, sediment grain size and the responses of other macrofaunal species may be as important as sea ice persistence in influencing pelagic-benthic coupling dynamics that lead to benthic community transitions.

A key question for the Pacific Arctic region is: how important are krill as prey for upper trophic level predators such as seabirds and whales? This requires investigating the more ephemeral prey aggregations in the epi-benthic and pelagic realm. The ICA presented here suggests that the offshore shift of gray whale distribution after 2015 may have been triggered by access to abundant krill in the northeastern Chukchi Sea. This work builds on research focused on bowhead whale (*Balaena mysticetus*) foraging in the western Beaufort Sea [28], which is complemented by multiple studies wherein ocean conditions associated with bowhead habitat selection have been used to infer their year-round reliance on finding krill as well as copepods and other zooplankton to feed on [60]. Seabirds can also indicate krill availability in the northeastern Chukchi Sea [61], especially short tailed shearwaters who rely on these advected prey now more than in the 1980s [62]. Using both marine mammal and bird species as ecosystem ‘samplers’ can provide clues to changes in the pelagic realm across a broad range of space and time related to each species natural history [22].

The AMP conceptual model provides a rudimentary framework guide for future research aimed at elucidating changes in both benthic and pelagic realms of the Pacific Arctic. In the benthic realm, a focus on linking sea ice metrics [10] to biophysical data will provide a

clearer picture of their combined influence on benthic communities [33]. The addition of sediment trap data will add additional detail for pelagic-benthic coupling in the northeastern Chukchi Sea [63]. In the pelagic realm, a pair of moorings (equipped with a 150 or 300 kHz ADCP and/or AZFP) deployed at ~70 m depth with one deployed near the offshore end of the DBO5 transect line and the other deployed near the offshore end of the DBO6 transect line would enable detection of signals associated with the diurnal vertical migration (DVM) of krill transiting the northeastern Chukchi Sea, while also monitoring the occurrence of overwintering krill [28]. Marine mammal and bird surveys in conjunction with the mooring-based DVM information can address foraging behavior plasticity of short tailed shearwaters and gray and bowhead whales in the northeastern Chukchi and western Beaufort Seas.

Acknowledgments

We thank the captains and crews of the research vessels that provided safe operations and space for a marine mammal observer on the DBO research cruises. We thank the passive acoustic team from AFSC and CICOES who manually analyzed data from recorders on moorings in DBO2 and DBO3: Eric K. Braen, Stephanie L. Grassia, Eliza G. Ives, Daniel F. Woodrich, and Dana L. Wright. We appreciate the marine mammal observers, pilots, mechanics, programmers, flight followers, and organizations that enabled ASAMM surveys to be conducted safely and effectively, in particular Megan Ferguson, Amelia Brower, Amy Willoughby, NOAA Aircraft Operations Center, and Clearwater Air, Inc. JMG thanks past and current support staff at CBL for technical assistance, especially Stephanie Soques and Alicia Clark, and Alynne Bayard for GIS mapping and data analytics.

References

1. Overland JE, Dunlea E, Box JE, Corell R, Forsius M, Kattsov V, et al. The urgency of Arctic change. Polar Sci. 2019 https://doi.org/10.1016/j. polar.2018.11.008.
2. Fetterer F, Knowles K, Meier WN, Savoie M, Windnagel AK. Sea Ice Index, Version 3. North Regional Sea Ice Monthly, updated daily. 2017 Boulder, Colorado USA. NSIDC: National Snow and Ice Data Center https://doi.org/10.7265/N5K072F8.
3. Polyakov IV, Aikire MB, Bluhm BA, Brown KA, Carmack EC, Chierichi M. Borealization of the Arctic Ocean in response to anomalous advection from sub-Arctic seas. Frontar Sci. 2020 7:491 doi: 10.3389/fmars.2020.00491.
4. Woodgate RA. Increases in the Pacific inflow to the Arctic from 1990 to 2015, and insights into seasonal trends and driving mechanisms from year-round Bering Strait mooring data. Prog Oceanogr. 2018 160:124-54.
5. Woodgate RA, Peralta-Ferriz C. Warming and freshening of the Pacific inflow to the Arctic from 1990-2019 implying dramatic shoaling in Pacific Winter Water ventilation of the Arctic water column. Geophy Res Lett. 2021 *48*, e2021GL092528. https://doi.org/10.1029/2021GL092528.
6. Danileson SL, Ahkinga O, Ashjian C, Basyuk E, Cooper LW, Eisner L, et al. Manifestation and consequences of warming and altered heat fluxes over the Bering and Chukchi Sea continental shelves. Deep-Sea Res II 2020 https://doi.org/10.1016/j.dsr2.2020.104781.
7. Duffy-Anderson JT, Stabeno P, Andrews AG, Kieciel K. et al. Responses of the northern Bering Sea and the southeast Bering sea pelagic ecosystems following record-breaking low winter sea ice. Geophys Res Lett. 2019 46 https://doi.org/10.1029/2019 GL083396.
8. Huntington HP, Danielson SL, Wiese FK, Baker M, Boveng P, Citta JJ. et al. Evidence suggests potential transformation of the Pacific Arctic ecosystem is underway. Nat Clim Chang. 2020 https://doi.org/10.1038/s41558-020=0695-2.
9. Lewis, KM, van Kijken GL, Arrigo R. Changes in phytoplankton concentration now drive increased Arctic Ocean primary production. Science. 2020 369:198-202, https://doi.org/10.1126/science.aay8380.
10. Frey KE, Young LNC, Comiso JC, Stock LV, Grebmeier JM, Cooper LW. Trends in sea ice cover, sea surface temperature, and chlorophyll biomass across the Distributed Biological Observatory in the Pacific Arctic region. this volume.
11. Nerini M. A review of gray whale feeding ecology. In: Jones ML, Swartz SL, Leatherwood S, editors. The Gray Whale *Eschrichtius robustus*. Florida: Academic Press; 1984. pp. 423-50.
12. Yablokov A, Bogoslovskaya LS. A review of Russian research on the biology and commercial whaling of the gray whale. In: Jones ML, Swartz SL, Leatherwood S, editors. The Gray Whale *Eschrichtius robustus*. Florida: Academic Press, 1984. pp.465-85
13. Brower AA, Ferguson MC, Schonberg SV, Jewett SC, Clarke JT. Gray whale distribution relative to benthic invertebrate biomass and abundance: Northeastern Chukchi Sea 2009-2012. Deep-Sea Res II 2017 144: 156-174 http://dx.doi.org/10.1016/j.dsr2.2016.12.00.

14. Bluhm BA, Coyle KO, Konar K, Highsmith R. High gray whale relative abundances associated with an oceanographic front in the south-central Chukchi Sea. Deep-Sea Res II 2007 54 (23-26): 2919-33.
15. Kim SL, Oliiver JS. Swarming benthic crustaceans in the Bering and Chukchi seas and their relation to geographic patterns in gray whale feeding. Can J Zool. 1989 67:1531–42.
16. Darling JD, Keogh IE, Streeves TE. Gray whale (Eschrichtius robustus) habitat utilization and prey species off Vancouver Island B.C. Mar Mamm Sci. 1998 14: 692-720.
17. Nelson TA, Duffus DA, Robertson C, Feyrer LJ. Spatial-temporal patterns in intra-annual gray whale foraging: Characterizing interactions between predators and prey in Clayquot Sound, British Columbia, Canada. Mar Mamm Sci. 2008 24(2): 356-70.
18. Hazen EI, Abrams B, Brodie S, Carroll G, Jacox MG, Savoca MC, Scales KI, Sydeman WJ, Bograd SJ. Marine top predators as climate and ecosystem sentinels. Front Ecol Environ. 2019 17 (10): 565-574, doi:10.1002/fee.2125.
19. Moore SE. Marine mammals as ecosystem sentinels. 2008 J Mamm. 89(3) 534-40.
20. Grebmeier JM, Moore SE, Cooper LW, Frey KE. The Distributed Biological Observatory: a change detection array in the Pacific Arctic – an Introduction. Deep-Sea Res II 2019 162: 1-7.
21. Moore SE, Logerwell E, Eisner L, Farley, Jr EV, Harwood LA, Kuletz K, Lovvorn J, Murphy J, Quakenbush LT. Marine fishes, birds and mammals as sentinels of ecosystem variability and reorganization in the Pacific Arctic region. In: Grebmeier JM, Maslowski W, editors. The Pacific Arctic Region-ecosystem status and trends in a rapidly changing environment. New York: Springer, 2014. pp.337-392.
22. Moore SE, Kuletz KJ. Marine birds and mammals as ecosystem sentinels in and near Distributed Biological Observatory regions: an abbreviated review of published accounts and recommendations for integration to ocean observatories. Deep-Sea Res II 2019 162: 211-217.
23. Moore,SE. Climate change. In: Wursig B, Thewissen JGM, Kovacs KM, editors. Encyclopedia of Marine Mammals, 3rd Edition London: Elsevier Academic Press, 2018. pp.194-197.
24. Berchok et al. Changes in timing and occurrence of marine mammals in the Northern Bering and Southern Chukchi Seas, 2012-2019. this volume
25. Clarke, JT, Brower, AA, Ferguson, MC, Willoughby, AL, Rothrock, AD. Distribution and Relative Abundance of Marine Mammals in the Eastern Chukchi Sea, Eastern and Western Beaufort Sea, and Amundsen Gulf, 2019. Annual Report, OCS Study BOEM 2020-027. 2020; Marine Mammal Laboratory, Alaska Fisheries Science Center, NMFS, NOAA, 7600 Sand Point Way NE, F/AKC3, Seattle, WA 98115-6349.
26. Moore SE, Haug T, Vikingsson GA, Stenson GB. Baleen whale ecology in Arctic and subarctic seas in an era of rapid habitat alteration. Prog Oceanogr. 2019 176:102-18. https://doi.org/10.1016/j.pocean.2019.05.010
27. Moore SE. Is it 'boom times' for baleen whales in the Pacific Arctic region? Biol Lett. 2016 http://dx.doi.org/10.1098/rsbl.2016.0251.

28. Ashjian, CJ, Okkonen SR, Campbell RG, Alatalo P. Lingering Chukchi Sea sea ice and Chukchi Sea mean winds influence population age structure of euphausiids (krill) found in the bowhead whale feeding hotspot near Pt. Barrow this volume
29. Moore SE, Stabeno PJ, Grebmeier JM, Okkonen SR. The Arctic Marine Pulses Model: linking annual oceanographic processes to contiguous ecological domains in the Pacific Arctic. Deep-Sea Res II 2018 152:8-21.
30. Christiansen F, Rodriguez-Gonzalez F, Martinez-Aguilar S, Urban J, Swartz S, Warick H, Vivier F, Bejder L. Poor body condition associated with an unusual mortality event in gray whales. Mar Ecol Prog Ser. 2021 658 237-252 https://doi.org/10.3354/meps13585
31. Burnham R, Duffus D, Mouy X. Gray whale (*Eschrictius robustus*) call types recorded during migration off the west coast of Vancouver Island. Front Mar Sci. 2018 5:1–11.
32. Grebmeier JM, Frey KE, Cooper LW, Kedra M. Trends in benthic macrofaunal populations, seasonal sea ice persistence and bottom water temperatures in the Bering Strait region. Oceanogr. 2018 31(2) https://doi.org/10.5670/oceanog.2018.224.
33. Grebmeier JM, Cooper LW, Goethel CL, Frey KE. Time series macrobenthic populations and sediment dynamic trends in the Northern Bering and Chukchi Seas this volume
34. Okkonen SR, Ashjian CJ, Campbell RG, Alatalo P. The encoding of wind forcing into the Pacific-Arctic pressure head, Chukchi Sea ice retreat and late-summer Barrow Canyon water masses. Deep-Sea Res II 2019 162: 22-31 doi.org/10.1016/j.dsr2.2018.05.00.
35. Okkonen S, Ashjian C, Campbell RG, Alatalo P. Diel vertical migration: a diagnostic for variability of wind forcing over the Beaufort and Chukchi Seas. Prog in Oceanogr. 2020 181, 2020102265 doi.org/10.1016/j.pocean.2020.102265.
36. Kalnay E, Kanamitsu M, Kistler R, Collins W, Deaven D, Gandin L, et al. 1996. The NCEP/NCAR 40-year reanalysis project. Bull Am Meteorol Soc. 1996 77: 437–70.
37. Berzin AA. Soviet studies of the distribution and numbers of the gray whale in the Bering and Chukchi seas, from 1968-1982. In: Jones ML, Swartz SL, Leatherwood S, editors. The Gray Whale *Eschrichtius robustus*. Florida: Academic Press; 1984. pp. 409-422.
38. Blokhin SA. Investigations of gray whales taken in the Chukchi coastal waters, USSR. In: Jones ML, Swartz SL, Leatherwood S, editors. The Gray Whale *Eschrichtius robustus*. Florida: Academic Press; 1984. pp. 487-510.
39. Heide-Jorgensen MP, Laidre KL, Litovka D, Jensen MV, Grebmeier JM, Sirenko BI. Identifying gray whale (*Eschrichtius robustus*) foraging grounds along the Chukotka Peninsula, Russia, using satellite telemetry. Polar Biol. 2012 35: 1035-1045 doi 10.1007/s00300-011-1151-6.
40. Braham HW. Distribution and migration of gray whales in Alaska. In: Jones ML, Swartz SL, Leatherwood S, editors. The Gray Whale *Eschrichtius robustus*. Florida: Academic Press; 1984. pp. 249-266.
41. Moore SE, DeMaster DP, Dayton PK. Cetacean habitat selection in the Alaskan Arctic during summer and autumn. Arctic 2000 53(4): 432-447.
42. Coyle KO, Bluhm B, Konar B, Blanchard A, Highsmith RC. Amphipod prey of gray whales in the northern Bering Sea: comparison of biomass and distribution between 1980s and 2002-2003. Deep-Sea Res II. 2007 54: 2906-2918.

43. Moore SE, Grebmeier JM, Davies JR. Gray whale distribution relative to forage habitat in the northern Bering Sea: current conditions and retrospective summary. Can J Zoo. 2003 81: 734-742.
44. Kędra M, Grebmeier JM. Ecology of Arctic shelf and deep ocean benthos. In: Thomas DN, editor. Arctic Ecology. Wiley-Blackwell 2021 pp. 325-355.
45. Kuletz K, Cushing D, Labunski E. Distributional shifts among seabird communities of the Northern Bering and Chukchi seas in response to ocean warming during 2017-2019. Deep-Sea Res II. 2020 181-182, doi.org/10.1016/j.dsr2.2020.104913.
46. Grebmeier JM, Barry JP. 1991. The influence of oceanographic processes on pelagic–benthic coupling in polar regions: a benthic perspective. J Mar Syst. 1991 2: 495–518.
47. Hill V, Ardyna M, Lee SH, Varela DE. Decadal trends in phytoplankton production in the Pacific Arctic Region from 1950 to 2012. Deep-Sea Res II. 2018 152: 82-94, doi.org/101016/j.dsr2.201812.015.
48. Waga H, Hirawake T, Grebmeier JM. Recent changes in benthic macrofaunal community composition in relation to physical forcing in the Pacific Arctic. Polar Biol. 2020 43:285–294 https://doi.org/10.1007/s00300-020-02632-3.
49. Grebmeier JM, Bluhm BA, Cooper LW, Danielson SL, Arrigo KR, Blanchard AL, Clarke JT et al. Ecosystem characteristics and processes facilitating persistent microbenthic biomass hotspots and associated benthivory in the Pacific Arctic. Prog Oceanogr. 2015 136: 92-114, doi.org/10.1016/j.pocean.2015.05.006.
50. Dauvin, J-C., L. Sampaio, A.M. Rodrigues, and V. Quintino. 2021. Taxonomy and ecology of sympagic *Ampelisca* species (Crustacea, Amphipoda) from the Strait of Gibraltar to the Strait of Dover, North-Eastern Atlantic. Front Mar Sci. 2021 doi.org/10.3389/fmars.2021.643078.
51. Pickart RS, Spall MA, Lin P, Bahr F, McRaven LT, Arrigo KR, Grebmeier JM. Physical controls on the macrofaunal benthic biomass in Barrow Canyon, Chukchi Sea. J Geophys Res Oceans. 2021 126, e2020JC017091 doi.org/10.1029/2020JC017091.
52. Stabeno P, Kachel N, Ladd C, Woodgate R. Flow patterns in the eastern Chukchi Sea. J Geophys Res Oceans. 2018 123 1177-1195 https://doi. org/10.1002/2017JC013135
53. Moore SE, Urban J, Perryman WL, Gulland F, Perez-Cortes H, Wade PR, Rojas-Bracho L, Rowles T. Are gray whales hitting 'K' hard? Mar Mamm Sci. 2001 17(4): 954-958.
54. Stimmelmeyr r, Gulland FMD. Gray whale (*Eschrichtius robustus*) health and disease: review and future directions. Front Mar Sci. 2020 doi: 10.3389/fmars.2020.588820
55. Stewart JD, Weller DW. Abundance of eastern north Pacific gray whales 2019-2020. NOAA-TM-NMFS-SWFSC-639 https://doi.org/10.25923/bmam-pe91
56. Suryan RM, Arimitsu ML, Coletti HA, Hopcroft RR, Lindeberg MR, Barbeaux SJ, Batten SD, et al. Ecosystem response persists after prolonged marine heat waves. Nature Sci Rep. 2021 https://doi.org/10.1038/s41598-021-83818-5.
57. Boveng PL, Ziel HL, Mc Clintock BT, Cameron MF. Body condition of phocid seals during a period of rapid environmental. Change in the Bering Sea and Aleutian Islands, Alaska. Deep-Sea Res II. 2020 181-182 https://doi.org/10.1016/j.dsr2.2020.104904.
58. Newell CL, Cowles TJ. Unusual gray whale *Eschrichtius robustus* feeding in the summer of 2005 off the central Oregon Coast. Geophys Res Lett. 2006 33 doi:10.1029/2006GL027189.

59. Feng Z, Rubao J, Ashjian C, Zhang J, Campbell R, Grebmeier GM. Benthic hotspots on the northern Bering and Chukchi continental shelf: spatial variability in production regimes and environmental drivers. Prog Oceanogr. 2021 https://doi.org/10.1016/j.pocean.2020.102497
60. Citta JJ, Quakenbush L, George JC. Distribution and behavior of Bering Chukchi-Beaufort bowhead whales as inferred by telemetry. In: George JC, Thewissen, JGM, editors. The Bowhead Whale *Balaena mysticetus*: biology and human interactions. London: Elsevier/Academic Press, 2021 pp. 31-56.
61. Gall AE, Day RH, Weingartner TJ. Structure and variability of the marine-bird community in the northeastern Chukchi Sea. Cont Shelf Res. 2013 67: 96-115 doi.org/10.1016/j.csr.2012.11.004
62. Kuletz K, Cushing D, Okkonen S, Ashjian C, Osnas E, Labunski E, Gall A. Short-tailed shearwater migrations through the Pacific Arctic. this volume
63. Laland C, Grebmeier JM, McDonnell A, Hopcroft RR, O'Daly S, Danielson S. Impact of a warm anomaly in the Pacific Arctic region derived from time-series export fluxes. this volume

Supporting Information

S1 Table: Summary of DBO cruises where a marine mammal watch was conducted

S2 Table: Summary of survey effort, gray whale sighting and calculation of sighting rate (SR) from ASAMM 2009-2019.

Figure

Click here to access/download;Figure;Fig 1.tiff

Figure 1

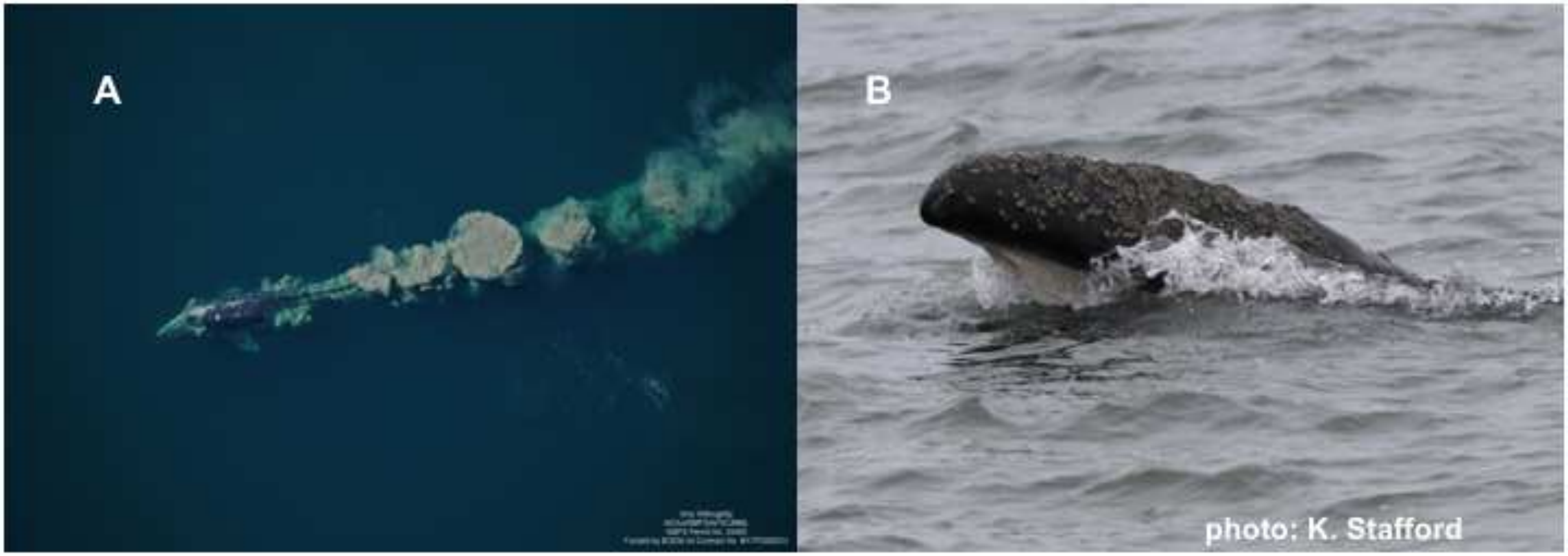

Figure Click here to access/download;Figure;Fig 2A.tiff

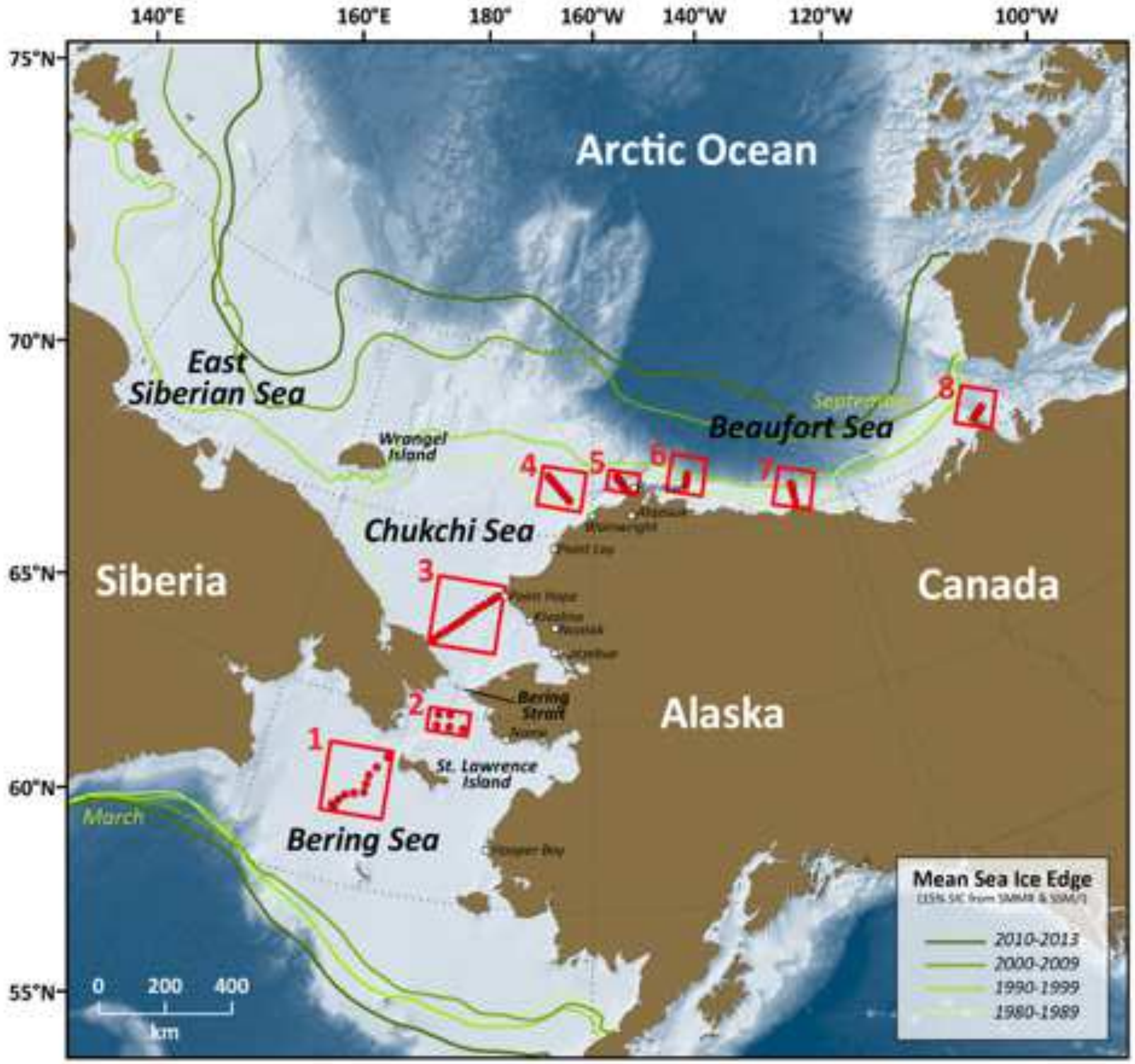

Figure 2A

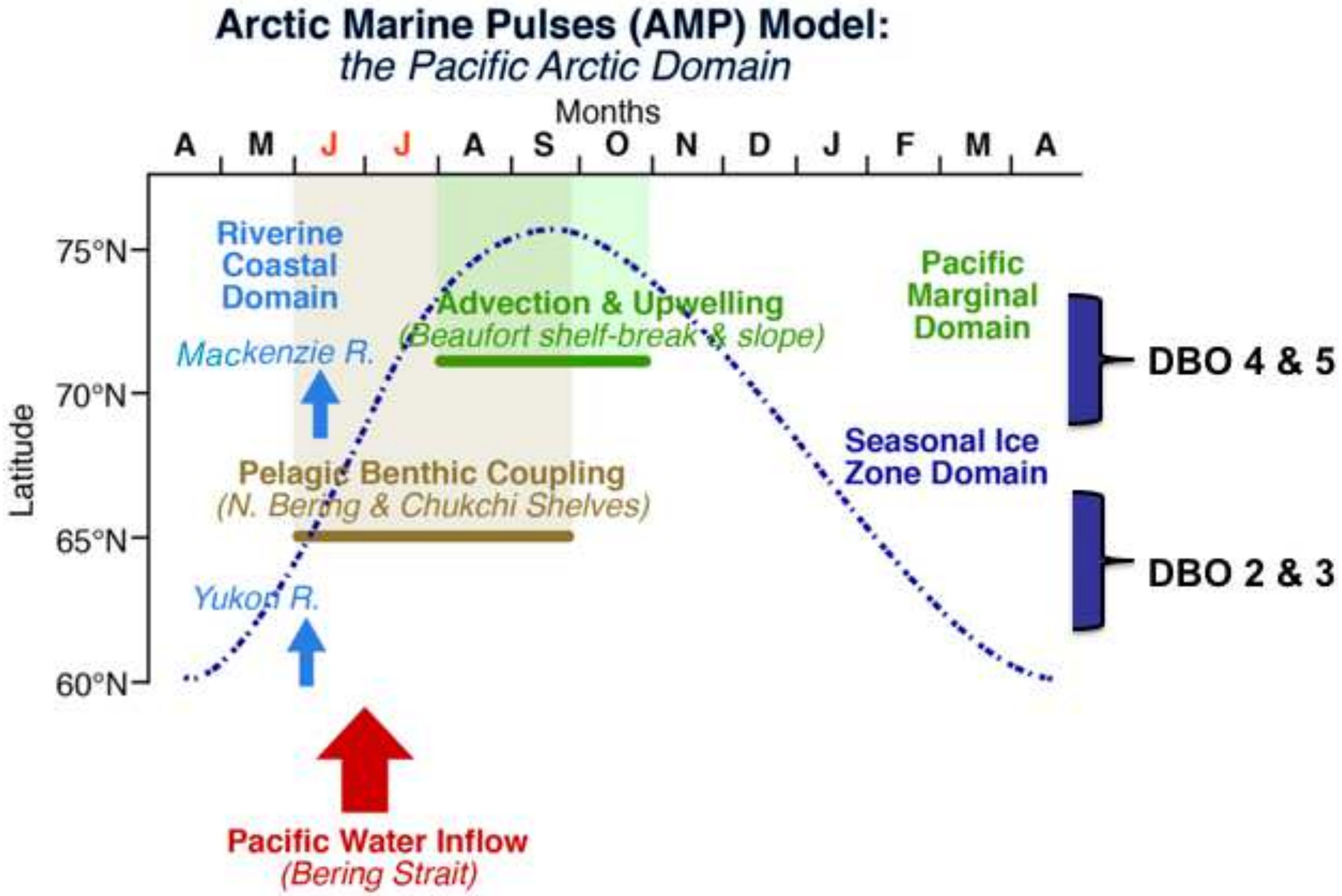
Arctic Marine Pulses (AMP) Model:
the Pacific Arctic Domain
Months
A M J J A S O N D J F M A
Latitude
75°N
70°N
65°N
60°N
Riverine Coastal Domain
Mackenzie R.
Yukon R.
Advection & Upwelling
(Beaufort shelf-break & slope)
Pelagic Benthic Coupling
(N. Bering & Chukchi Shelves)
Pacific Marginal Domain
Seasonal Ice Zone Domain
DBO 4 & 5
DBO 2 & 3
Pacific Water Inflow
(Bering Strait)

Figure 2B

Figure 3

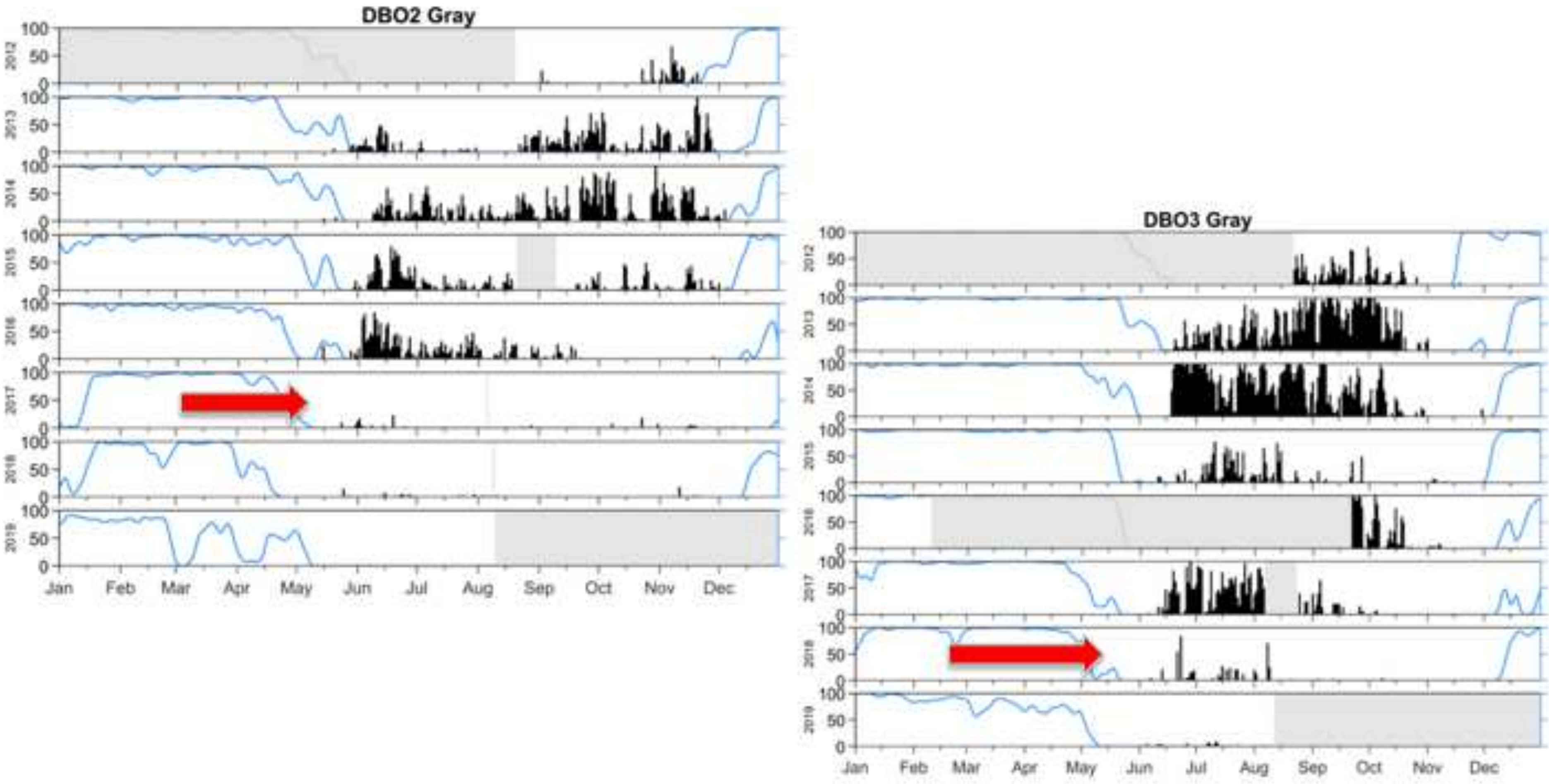
DBO2 Gray
DBO3 Gray
100
50
0
2012
2013
2014
2015
2016
2017
2018
2019
Jan
Feb
Mar
Apr
May
Jun
Jul
Aug
Sep
Oct
Nov
Dec

Figure

Click here to access/download;Figure;Fig 4A.tiff

Figure 4A

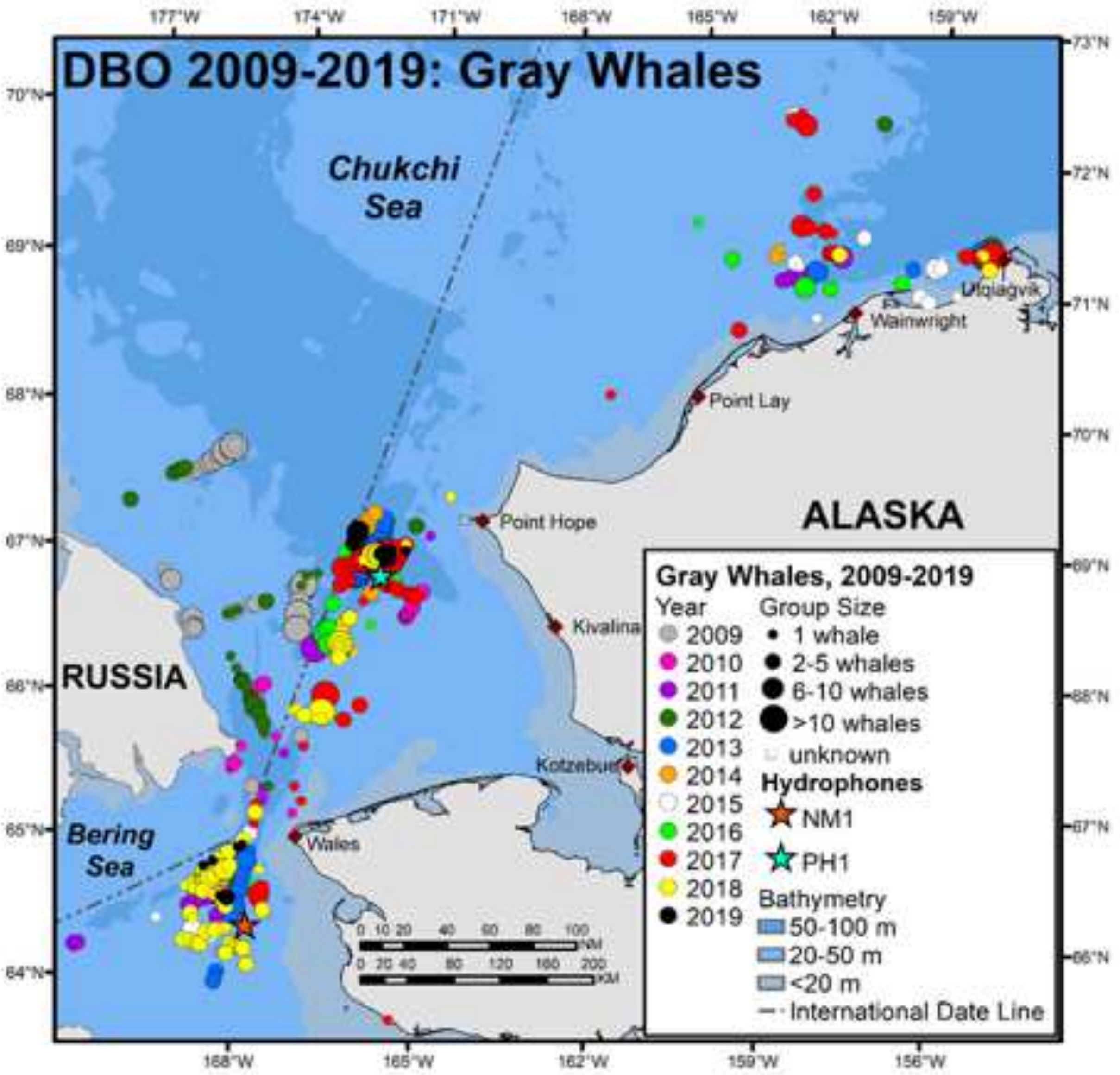

Figure Click here to access/download;Figure;Fig 4B.tiff

Figure 4B

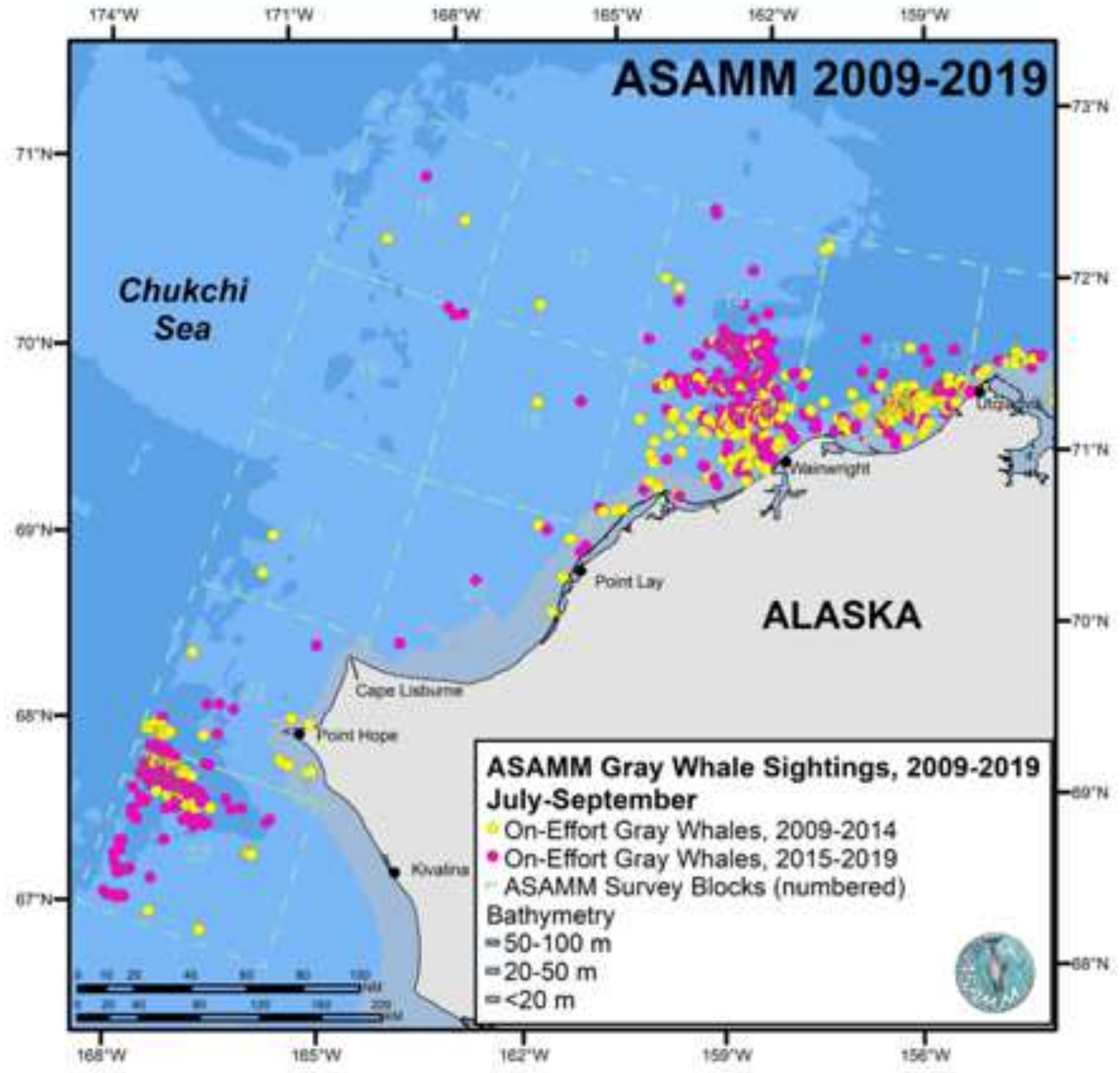

Figure

Click here to access/download;Figure;Fig 5.tiff

Figure 5

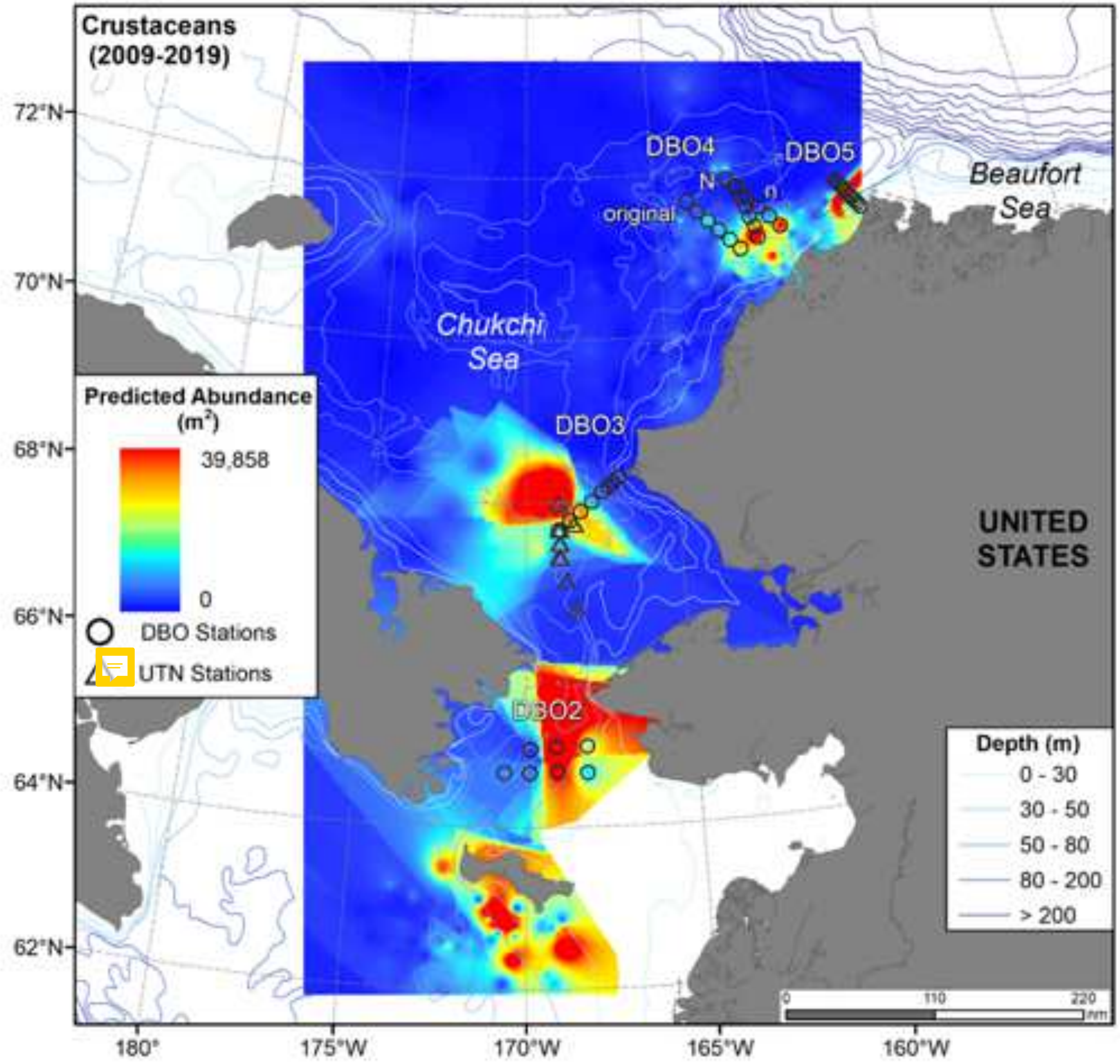

Click here to access/download;Figure;Fig 6.tiff

Figure 6

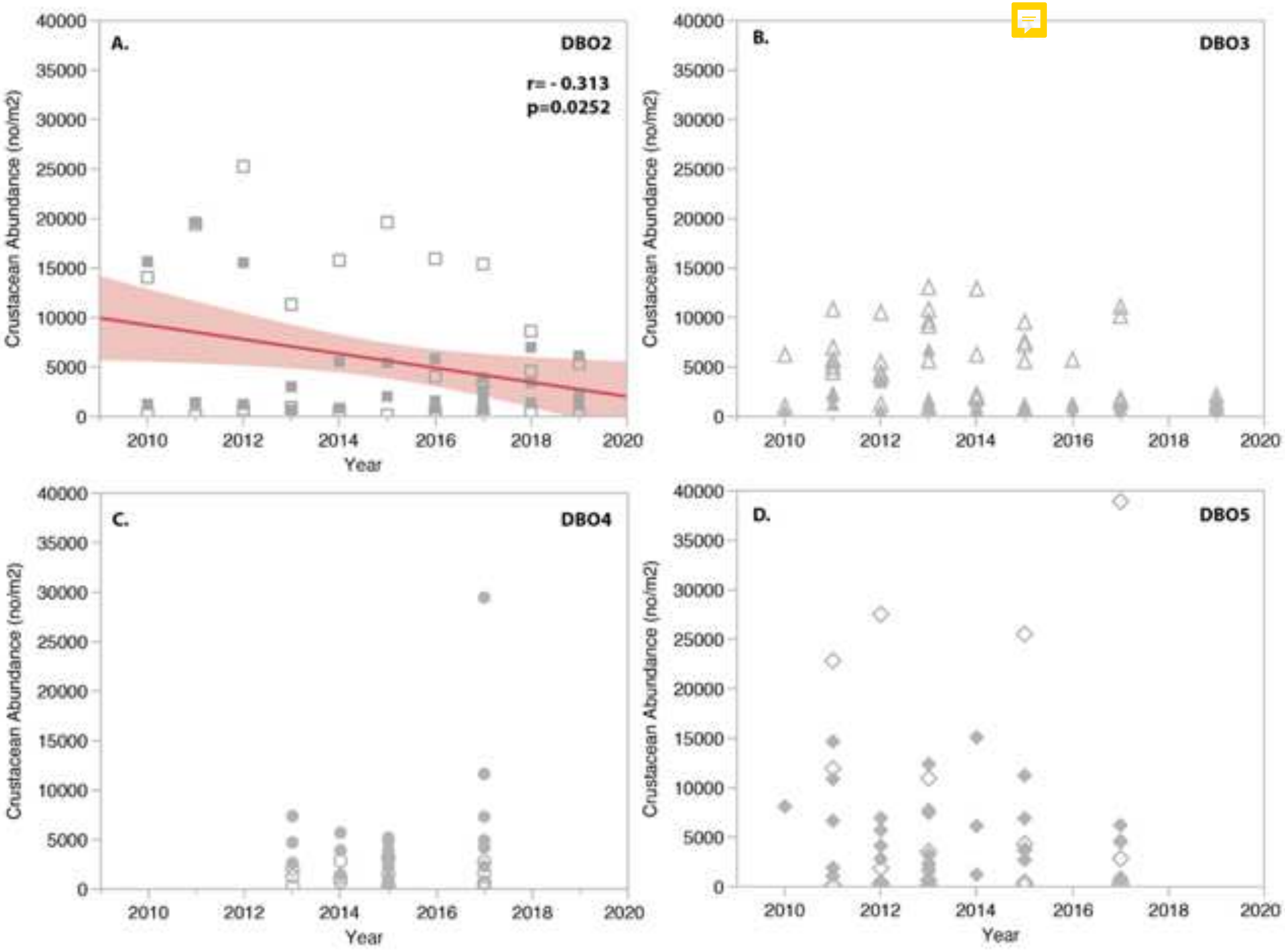

Figure

Click here to access/download;Figure;Fig 7.tiff

Figure 7

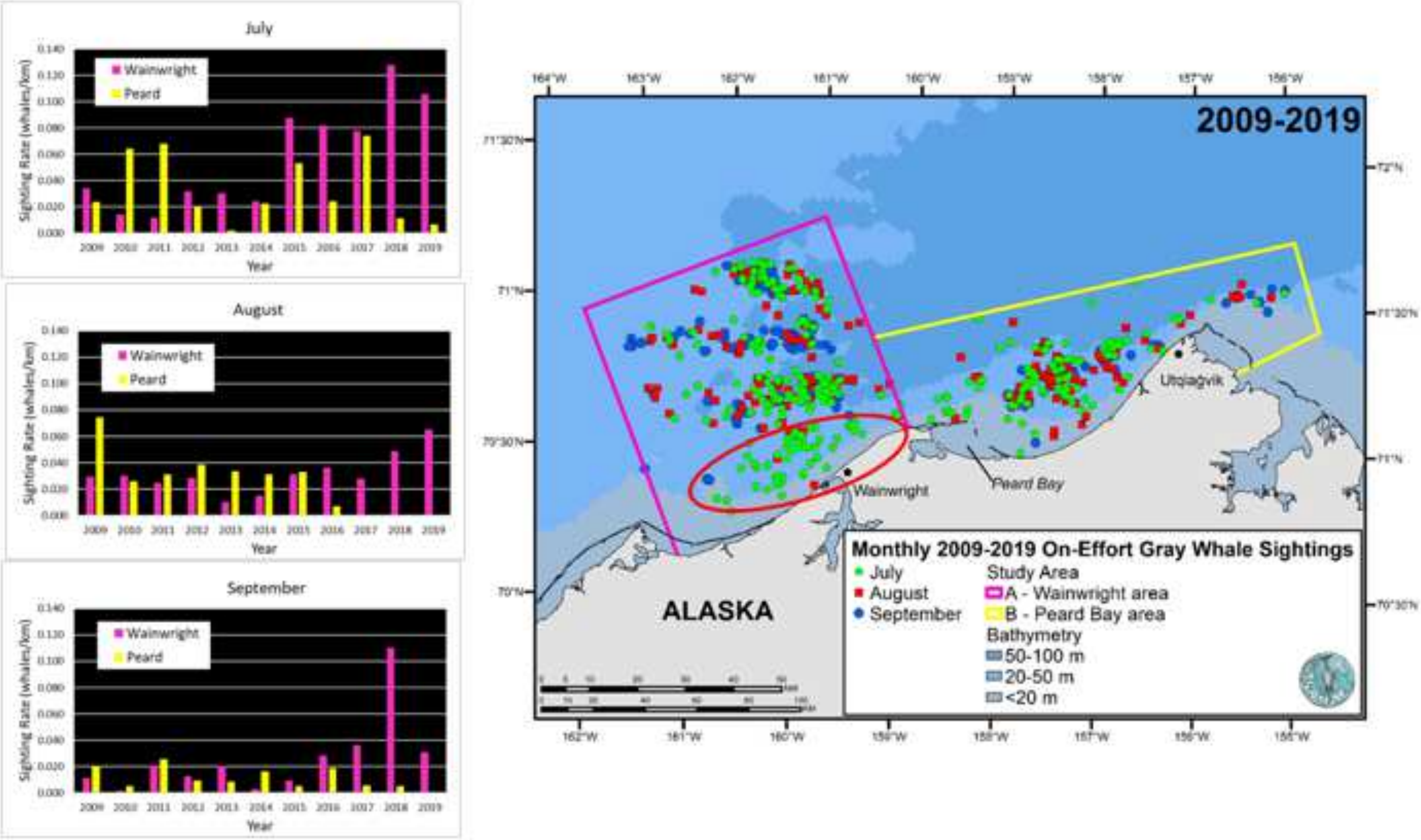

Figure

Click here to access/download;Figure;Fig 8.tiff

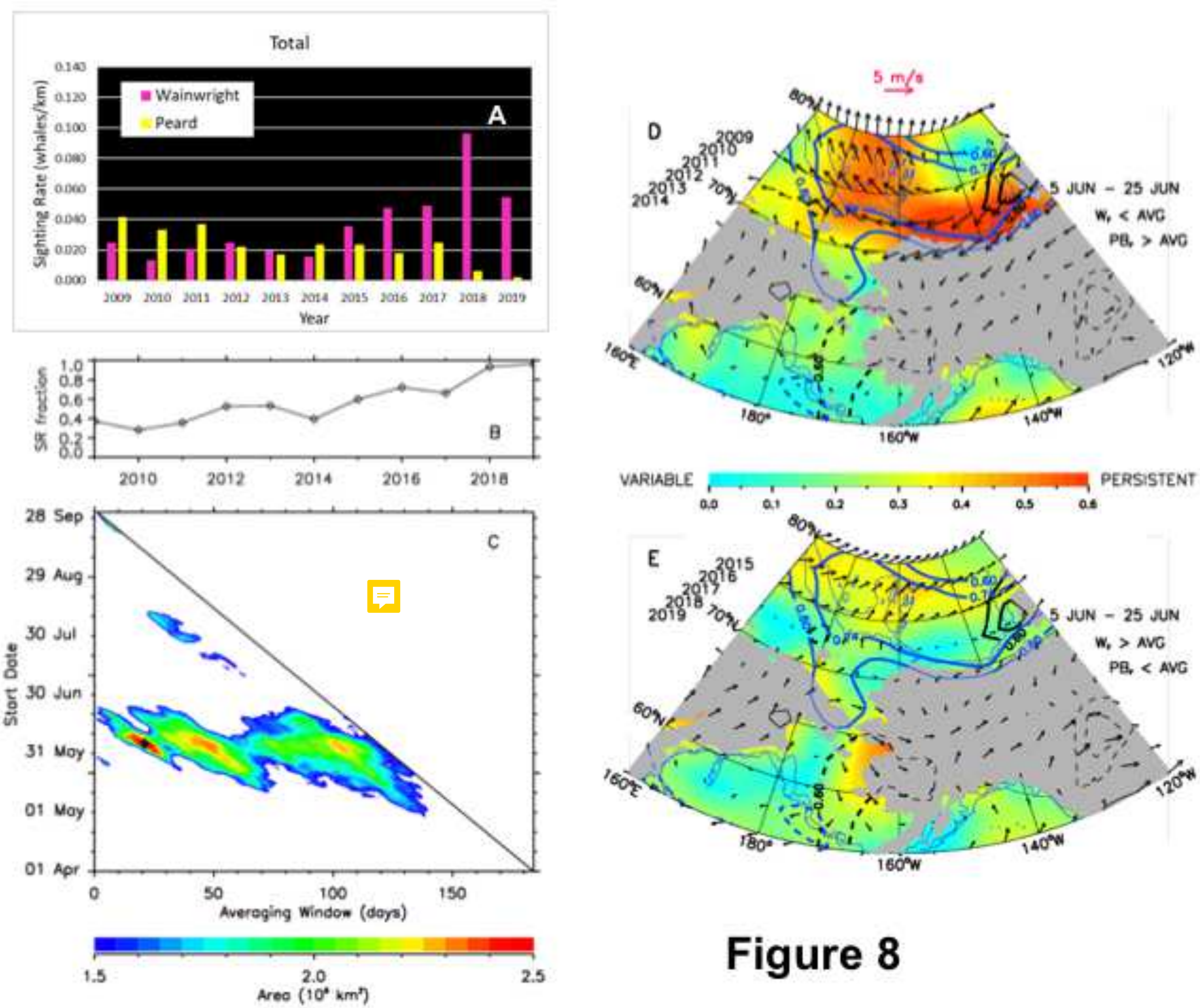

Figure 8

Figure

Click here to access/download;Figure;Fig 9.tiff

Figure 9.

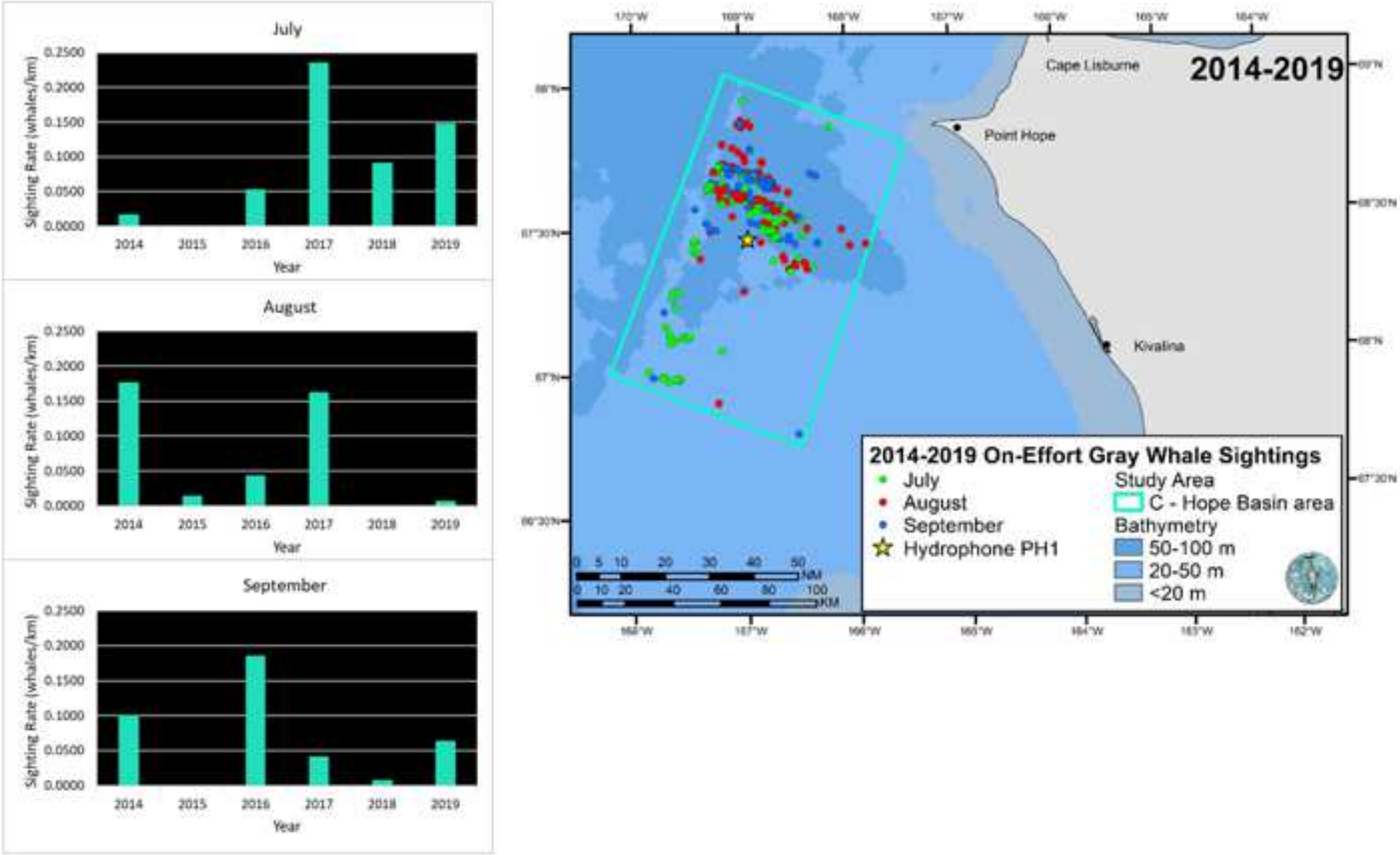

Figure 10

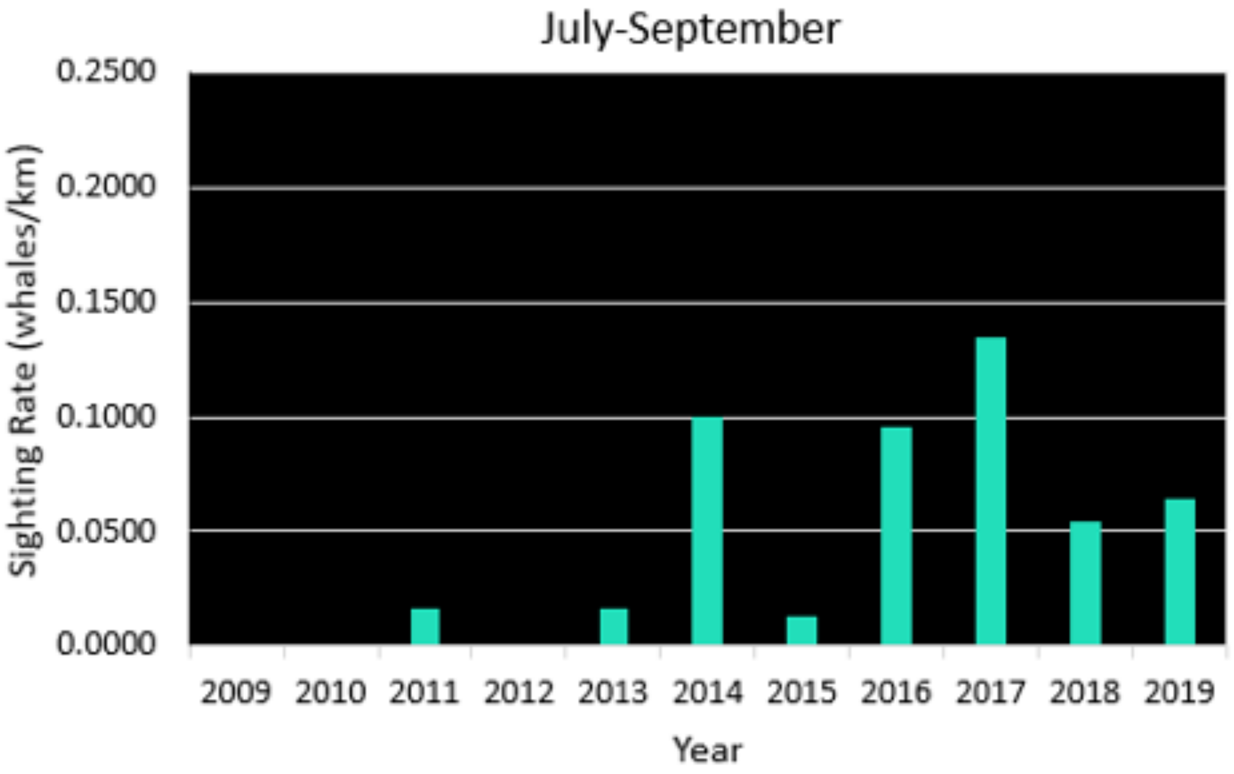
July-September
0.2500
0.2000
0.1500
0.1000
0.0500
0.0000
Sighting Rate (whales/km)
2009
2010
2011
2012
2013
2014
2015
2016
2017
2018
2019
Year

Click here to access/download
Supporting Information
GW_SupplementalTables.docx
