# Supplementary material for: Changes in gray whale phenology and distribution related to prey variability and ocean biophysics in the northern Bering and eastern Chukchi seas
Source: PLoS One. 2022 Apr 7;17(4):e0265934. doi: 10.1371/journal.pone.0265934 (PMC8989348; doi:10.1371/journal.pone.0265934)
Supplement: Attachment — Submitted filename: Response to Reviewers.docx [file pone.0265934.s005.docx]

**PONE-D-21-20781**

**Changes in gray whale phenology and distribution related to prey variability and ocean biophysics in the northern Bering and eastern Chukchi seas**

RESPONSE TO REVIEWERS (in blue, line numbers = as in Manuscript with Track Changes)

**Reviewer #1:** This manuscript compiles some excellent, longitudinal data on gray whale sightings, prey distribution and availability, and oceanography in the Arctic region that was collected synchronously. It is a very impressive dataset and the authors should be commended in their field efforts to collect these data. The questions addressed revolve around the changing oceanography in the Arctic region and impacts on prey availability and gray whale distribution over the sampling period. This research is very interesting, and answers are needed to better understand the impacts on such a large population of crustacean predators (gray whales) and the overall ecosystem response to rapid environmental change. In particular, I feel the changes in acoustic, oceanography and prey data over time and space are interesting and solid (Fig. 3, Fig 6, Table 2), but I feel the links to gray whale spatial distribution patterns are not well connected or presented.

While the data are impressive, I found the spatial analysis to be lacking in adequate description and standardization, which makes assessment of results and author interpretation challenging. In particular, the survey effort conducted during DBO cruises needs better description and evaluation. Presentation of raw numbers of sightings is misleading without standardization of this information by the amount of survey effort (i.e., km).

DBO sighting data were obtained during a standardized watch for marine mammals on DBO cruises that followed a similar track each year, but variability in number of watch standers and ship operations confounded quantification of watch effort. Therefore, DBO sighting data were NOT used in statistical analysis, as was stated in the Methods of the original submission. We now emphasize the distinction between DBO sighting data and ASAMM sighting data where effort is available from which to calculate sighting rates (i.e. whales/km).

Examples of specific revisions in this regard include:

a. Methods: lines 187-190; 212-2014
b. Supplementary tables for each data set; i.e. **Table S-1** DBO cruise summary and Table **S-2** ASAMM survey data including survey effort and gray whale sighting information
c. Results: lines 343-351, Fig 4 caption and Table 1

Additionally, the authors discuss overlap between areas of high whale sightings and different prey availability, but this analysis is not described and results not displayed. This overlap analysis could be highly enlightening but needs more details and clarity.

We do not use the term ‘overlap’ in the paper and any ‘overlap analysis’ would require some type of modeling to adjust for the very different spatial & temporal sampling regimes represented by visual surveys from a moving vessel/aircraft and sediment grabs repeated at specific locations along a set transect. We feel maps presenting gray whale distribution (Fig 4) and infaunal crustacean abundance (Fig 5) are the best way to show convergent areas of relative abundance for large free-swimming predators re. sessile-benthic prey.

Furthermore, I am unclear how figure 5 was produced. It appears to be a GIS interpolation of crustacean sample data, but the input data is not described, nor the analysis methods. There are many types of interpolation methods, all of which have bias and assumptions, so its important to clearly describe the approach used.

Specifics on how Figure 5 was produced are now given in Methods (lines 256-272), with additional details in Results (lines 405-427) and the Fig 5 caption. Details on exactly what data went into the mapped abundance are provided, by station, cruise and year in Supplementary Table 3 (**Table S-3**).

Overall, I feel that more details on statistical methods are needed. Some results are presented that were not described in the methods section. Additionally, I think there is a need to link the research questions more clearly to the statistical analyses so that the work appears more directed and purposeful.

Additional details on statistical methods are provided, as noted in the response above re. Fig 5 and in responses below, re the ICA method. Methods are provided for all results presented.

The reviewer may have missed the point that this paper does not report on the results of a gray whale-focused long-term study (please see lines 139-149). Specifically, this paper presents a *compilation* of data on: (a) gray whale phenology and distribution from long-term datasets, combined with (b) long-term records of infaunal crustacean prey abundance and (c) application of the ICA method to provide a modeling approach to assess krill availability (similar to ICA in Ashjian et al. 2021, published as part of the Plos ONE DBO-Special Collection. The work of compiling and synthesizing these observations and methodologies was indeed ‘directed and purposeful’ and we hope our results garner enough interest that some directed hypothesis-driven research might be supported in the future.

Also, the authors split the data set temporally at 2014/15 or 2013/14 for different analyses and no justification is provided for selecting these dividing points, or explanation of why different times spans are analyzed for different data.

We think this comment results from confusion about the ICA (see next comment). As explained in Methods (lines 273-285) and noted in Results (lines 610-6401 and Fig 9) the correlation of variability in regional winds &/or transport with gray whale abundance is based on calculation of annual sighting rates (SR = whales/km) for data from July-August-September in each year. Data were not ‘split’ into two (or more) periods, rather the annual SRs in the 3 analytical areas serve as a training set (see line 613 Fig 9B) for the iterative correlation with transport (at mooring A3) and/or regional winds.

The ICA analysis seems like an interesting and robust method of time-series analysis but this is new to me so I cannot fully evaluate the approach or results. I did find the results hard to interpret so perhaps the results need more “hand-holding” for people like me who are unfamiliar with this method.

We tried to simplify wording in both the ICA method and the results sections of the ms.; please see Lines mentioned above. Also, please note that Okkonen et al. 2019 & 2020 [35 & 36] provide additional detail on the ICA method. We also provide specific reference to the application of the ICA in the Plos ONE Ashjian et al. 2021 paper [28], which describes variability in krill availability to bowhead whales in waters adjacent to the study area in this paper.

Discussion of gray whale foraging on krill, and the driving role of oceanography, is confusing to me as the authors do not present any data on krill collection or analysis, as far as I can tell (krill are not listed in Table 2). The reference to the Grebmeier manuscript in this same volume makes it hard to know what taxa were collected and included in this analysis. And the paper that is referenced regarding gray whale foraging on krill (Bluhm et al.) is in the south-central Chukchi Sea, which is not the same area that is discussed in this paper (DBO regions 4 and 5 in the northeastern Chukchi Sea). The Kim and Oliver paper do not mention euphausiids. I understand that the potential role of krill as a target species for foraging Arctic gray whales is emerging, so data is minimal, but I think it needs to be more clear that this new foraging strategy is still speculation due to the paucity of data.

Not sure how to respond to this overarching comment, as the Reviewer has everything “right”.

Indeed, no krill data are available for presentation in this paper and we state that we have no direct evidence that gray whales are actually consuming krill (e.g. lines 1000-1002). We feel we were very clear that gray whales have been observed skim feeding krill near bowheads feeding on krill swarms in waters east of our study area (Fig 1B and caption). We also clearly state that feeding gray whales were associated with krill patches in the southern Chukchi Sea/DBO 3 area, as reported by Bluhn et al.. We cite the Kim and Oliver paper for their report of gray whales feeding on cumaceans, not krill. And near the end of the Discussion (lines 1143-1145) we point out that because we have no direct evidence (feces or stomach contents) that gray whales are feeding on krill our findings are speculative and the importance of krill to gray whales and other species should be the focus of future research.

The authors are clearly very knowledgeable about the ecology and oceanography of this region, and some ideas presented are very interesting, but I found the Discussion to be too long with a fair bit of speculation and assumptions based on ‘noteworthy’ ideas. Additionally, there were a few parts of the results and discussion sections that seemed redundant (same information presented or stated) so I think the Discussion can be trimmed down and written in a more concise and precise manner, with less speculation. I suggest the authors focus on the hard, supported conclusion their analysis reveals.

We edited the Discussion eliminate redundancy and make the presentation more concise; please see text deletions and track-changes throughout the section.

I found some of the figures hard to fully interpret due to a lack of full legends, description, and clarity. I think it’s also important to recognize that many readers (myself included) are not intimately familiar with the Artic region and the DBO project (sampling scheme, acronyms, etc.). So, any effort to improve labels and descriptions of methods would be helpful.

Many of the Figures were revised to ensure place names used in the text are mapped. Font sizes were enlarged to improve readability and Figures and captions were revised to include additional detail explaining results and specifying what data were used to construct the figure and where that data can be accessed (also see Supplemental Tables).

I provided many specific comments in the manuscript PDF regarding my questions and suggestions for improvements. I sincerely hope my feedback helps improve the analysis and presentation of the results so that the readers can see and understand the author’s interpretations and conclusions better. There is high value in this research, and I am excited to see the authors publish their study, but I feel that the analysis needs more attention and description.

We responded to all the specific comments imbedded in the PDF copy of the ms., many of which re-stated or elaborated on or the general comments listed above. We sincerely thank Reviewer #1 (Dr. Leigh Torres) for her interest in our paper and the obvious time and care she took with the review; our public thanks to her appears in the Acknowledgments.

**Reviewer #2**: This paper documented how the gray while phenology and distribution change seasonally and interannually, and provided the potential drivers, including hydrographic and biological properties, ice condition and wind forcing. I am particularly interested in the section where the authors investigated the impact of wind on the geological shift of the gray whale SR near the head of the Barrow Canyon.

The authors suggested that gray whale distributes more in the Peard Bay area when persistent northeasterly wind blows, and more in the Wainwright area when the wind is weak and variable. I am curious the mechanism. The northeasterly wind can induce upwelling in the canyon which may in turn brings deep water onto the shelf, particularly in the coastal region. It seems to be consistent with the gray whale distribution in the Peard Bay which is confirmed in the coastal region (Fig. 7 -now Fig 8). By contrast, in the any other wind condition, the Alaskan Coastal Current flows off the shelf via the canyon, with a pumping-down process (Pickart et al., 2021 [64]). I suspect this might be the reason that in this wind regime, gray whale has a broader distribution in the Wainwright area where is still shallow and food-rich.

While we appreciate this detailed explanation, we note that we cited Pickart et al. 2021 in the original paper (now ref [64]) and while we generally agree with Reviewer #2 we feel our text describing the specific results of the ICA is the most appropriate for our paper and is, in fact, less speculative than the text above.

I understand the physical mechanisms are not the scope of the paper, but think it would be nice to briefly discuss these as the wind/hydrographic data have been presented. I also strongly recommend the authors to use high-res figures. Many figures are far from clear, e.g. the labels in Fig. 3, the years of the bar plots in Fig. 7- now Fig 8, the labels on the maps of Fig. 8 – now Fig 9.

We have revised figure labels, increased font size and clarified labeling, as described above in response to Reviewer #1..

Over all, the paper is informative with such great datasets and improves our understanding of gray whale in the Bering and Chukchi Seas. I would thus recommend a minor revision.


The specific comments and questions:

Some place names are not shown, e.g. Hope Basin, Pt. Franklin, Pt. Barrow (be sure everybody knows that it is Utqiaġvik on the maps – actually Pt Barrow is still the correct name, the village of Barrow changed to Utqiaġvik ). The Wainwright and Peard Bay were first shown prior to the results, although they have been presented in Fig.7. I would suggest to add the names on the Fig.2.

Names (including Utqiaġvik) were added to Fig 2A, Fig 4A as well as other maps. The boundaries of the Wainwirght, Peard Bay (Fig 8) and Hope Basin (Fig 10) analytical areas are (and were) clearly shown.

Label the two panels in Fig.3 A and B. I guess the blue curves are the ice concentration in somewhere. Please make it clearer in the caption.

Figure 3 labels were added and caption edited for clarification.

If I understand correctly, in Figure 5, the abundance map was interpolated using the data from the DBO and UTN stations shown in the figure. The authors need to remove the data away from the stations, which I think are fake and misleading.

The data used in Figure 5 and methods of interpolation used in map preparation are now clearly presented, as summarized earlier in response to Reviewer #1. There were no fake or misleading data presented, and now supplementary Table S-3 clarifies exactly what crustacean data are presented in this paper.

Table 2 is out of the page, I cannot see the column of DBO5.

Table 2 has been uploaded as a separate file (using the Other tab) to insure it’s horizontal orientation is retained.

Line 336. remove the bracket in front of the equation.
Done

What does the ellipse in Fig. 7 – now Fig 8 represent for?

The ellipse has been removed.

The caption of Fig. 8 – now Fig 9 is not clear. What does the color in the maps of Fig. 8. Variance? If so, variance of what? Wind speed? Or any wind component? What are the blue contours? Is the mean atmospheric circulation actually the composite of the 10-m wind?

The Figure 9 caption has been expanded to clarify the color shading and specify statistical meaning associated with the blue and black contours.

The summary in the Table 4 is very helpful!
