# Supplementary material for: Changes in gray whale phenology and distribution related to prey variability and ocean biophysics in the northern Bering and eastern Chukchi seas
Source: PLoS One. 2022 Apr 7;17(4):e0265934. doi: 10.1371/journal.pone.0265934 (PMC8989348; doi:10.1371/journal.pone.0265934)
Supplement: Attachment — Submitted filename: Response to Reviewer Comments on R1.docx [file pone.0265934.s006.docx]

**6. Review Comments to the Author**

Please use the space provided to explain your answers to the questions above. You may also include additional comments for the author, including concerns about dual publication, research ethics, or publication ethics. (Please upload your review as an attachment if it exceeds 20,000 characters)

**Reviewer #1 (Leigh Torres):** I appreciate the revision of this manuscript. I could follow how the data were used and applied to answer different questions much better than previously. I have some remaining comments below that I think the authors should address, but these should not be onerous and will help with clarity. I think this paper does a nice job of bringing together many datasets in a biologically complex region undergoing environmental change to reveal some patterns, highlight some unexpected findings, and pose interesting hypotheses worth follow up research. Great work. [Of note, I could never download or view Table 2. This was not included in the PDF provided for review. It is highly referenced in the text, so its unfortunate I could not review it.]

Fig 2 legend: Describe what the green and white lines are. Which are describing what?

Additional detail was added to figure caption.

Line 157: Change “distribution” to “presence”, since distribution includes understanding where animals are not and this assessment has not been conducted. “…sightings from DBO cruises provide data on gray whale presence,…”. I appreciate the clarification on the use (and non-use) of these data.

The word distribution changed to ‘presence’ (see line 161)

Line 171: You say three geographic areas but only 2 are listed. I am confused.

The three areas now clearly defined (see line 178)

Line 200-201: Change “Spatial analysis” to “spatial interpolation” in this sentence: “Spatial interpolation was accomplished using geographical information system software.”

The word analysis was changed to ‘interpolation’ (see line 214)

The data were not actually analyzed using the IDW, but rather interpolated for visual assessment. The Spearman’s rho rank correlation did the analysis part. I do appreciate the added details on the IDW method. Thanks.

Thank you for the improved description of what the ICA does and tests.

Visual detections section – This is much improved to clarify interpretation and limitations of data. For Figure 4, can you add the DBO areas? You refer to these areas in the text (lines 257-259) so I found myself awkwardly bouncing between the text, Figure 2A and Figure 4A to understand what was described. Addition of the DBO boxes would also help understand where data was collected and where it was not, since this is mainly presence-only data.
Line 275-277: I just don’t see this shift clearly in the data. Maybe because of overlapping data points. I suggest making the points more transparent, or using a kernel density approach to describe these shifted distribution patterns.
DBO regions now delineated by dashed lines in Figure 4A and analytical areas now delineated by dashed lines in Figure 4B. The figure caption was revised to include this information.

NOTE: The shift in distribution evident by the pink-yellow colors are quantified in sighting rate histograms provided in Figures 8 & 9.

Line 277-279: Are you refereeing to the west of Hope Point? Can you specify this? Otherwise I am not sure where the “trough” is.

The words ‘southwest of Pt Hope’ added for clarification (see line 297)

Line 281: Can you remind the reader of where these data were derived from? “with reference to observed changes in infaunal crustacean prey abundance and community composition derived from…”.

Added ‘from benthic sampling’ (line 301) and ‘from ASAMM data’ (line 302) to remind the reader of where data were derived from

Figure 5: resolution of this figure is still low so I can’t see the “closed gray circles” well. Also, I am unclear about the source of the “all stations”. Are these from the Arctic Data Center? Can you clarify?

Figure 5 was revised with higher resolution to improve clarity of presentation. Details of all station data availability now appear in the caption.

Figure 6: Please describe the red line for DBO 2. Is it for the open or closed circles? Or both?

Details regarding the meaning of symbols and the red regression line now appear in the revised caption for Fig 6.

Figure 7**:** This is a handy figure. Thanks for adding. But can you describe how these abundance time series visualizations were generated? I made have read it in the methods, but forgot by the time this figure came up. I think if you just add it to the legend that would help me.

We have revised text in the Methods (lines 198-204) to further describe how abundance visualizations were generated and guide the reader to that section in the revised caption for Fig 7. We do not think repeating methods text in the figure caption is appropriate

Can you plot Hanna Shoal on Figure 5?

Yes - Hanna Shoal call out (w/ arrow) was added to Figure 5

I think you should mention the scale of Fig 5? That it is a decadal view of crustacean abundance.

Figure 5 is (and was) clearly labeled as 2010-2019 at the top left; this period is now also specified in the revised figure caption.

As Fig 6 shows, this abundance is dynamic, so the scale of the data representation should be made clear.

The time scale is clearly represented along the X-axis, and is the same (2010-2019) for all crustacean prey figures.

Line 339: Where do these observations of “feeding gray whales” come from? ASAMM? I think it would be good to describe.

Added ‘from both vessel and aircraft platforms’, to clarify “feeding gray whales” statement (see line 364-365)

Line 437-438: Did you conduct a ICA analysis to determine this negative result of “no clear relationship”? This should be explained and detail. It strengthens the results show in **Fig 9.**

Yes, an ICA was conducted and no clear relationship found for the Hope Basin area; see lines 502-503.

Does the ICA account for interactions between the predictor variables? I would guess that volume, heat and freshwater transport interact with each other (likely in a non-linear way). Can you add a description into the methods about if/how ICA deals with those interactions?
As employed in this paper, ICA does not account for interactions between predictor variables.

The ICA method returns two temporal results: the start and end dates defining a period (alternately, a duration and lag) over which a time-averaged predictor variable is correlated with the gray whale sighting rate. In other words, ICA provides phenological information on relationships between gray whales and aspects of their environment.

The ICA method could potentially be adapted to account for interactions between multiple predictor variables, but additional iterative loops would be necessary to compute the interactions across the array of possible start/end dates for each the predictor variables. Depending on the number of predictor variables and the time domain to be investigated, the computational time (on a personal computer) could be many hours to days. We are skeptical that this level of analytical complexity would provide meaningful information beyond the results presented in this paper.

Line 497-498: The percentages add up to more than 100%. So what do they represent?

The represent percentage of stomach sample; as clarified in line 568=569

Line 529: Can you remind us when these DBO cruises were? Because the point here is that sightings of gray whales continued to be made during the DBO cruises even when the acoustic detections dropped. So, adding the overlapping time frame component to this sentence would be helpful.

Added ‘July-October’ to clarify DBO cruise timing (see line 602-603)

Thanks for the acknowledgement.

**Reviewer #2: (No Response)**
